# Synthesis, Characterization, and Preliminary In Vitro Anticancer Activity of Zinc Complexes Containing Amino Acid-Derived Imidazolium-Based Dicarboxylate Ligands

**DOI:** 10.3390/ijms26073202

**Published:** 2025-03-30

**Authors:** Carlos J. Carrasco, Antonio Pastor, María del Mar Conejo, Eleuterio Álvarez, José Manuel Calderón-Montaño, Miguel López-Lázaro, Agustín Galindo

**Affiliations:** 1 Departamento de Química Inorgánica, Facultad de Química, Universidad de Sevilla, 41071 Sevilla, Spain; ccarrasco1@us.es (C.J.C.); anpastor@us.es (A.P.); mconejo@us.es (M.d.M.C.); 2 Instituto de Investigaciones Químicas, CSIC-Universidad de Sevilla, Avda. Américo Vespucio 49, 41092 Sevilla, Spain; ealvarez@iiq.csic.es; 3 Departamento de Farmacología, Facultad de Farmacia, Universidad de Sevilla, 41012 Sevilla, Spain; jcalderon@us.es (J.M.C.-M.); mlopezlazaro@us.es (M.L.-L.)

**Keywords:** zinc, anticancer, selectivity, amino acid, imidazolium-dicarboxylate, X-ray

## Abstract

Coordination polymers containing zinc and imidazolium-based dicarboxylate ligands, [L^R^]^−^, were synthesized by reacting zinc acetate with HL^R^ compounds, **1**. The resulting complexes were characterized and structurally identified using single-crystal X-ray diffraction, revealing polymeric structures for the complexes [Zn(L^R^)_2_]_n_ (R = Gly, **2a**; βAla, **2b**) and [Zn(L^Leu^)_2_(H_2_O)_2_]_n_ (**2c**). In these structures, the [L^R^]^−^ ligands adopt a bridging monodentate μ-κ^1^-O^1^,κ^1^-O^3^ coordination mode, resulting in distorted tetrahedral (**2a**, **2b**) or octahedral (**2c**) geometries around the zinc center. When the synthesis was carried out in the presence of amino acids, mixed ligand complexes [Zn(L^R^)(aa)(H_2_O)]_n_ (R = aa = Val, **2d**, and R = aa = Ile, **2e**) were formed. Complexes **2d**–**2e** were also structurally characterized using single-crystal X-ray crystallography, revealing that the ligand [L^R^]^−^ maintained the same coordination mode, while the zinc center adopted a five-coordinated geometry. The cytotoxic activity of complexes **2a**–**2e** was evaluated against three cancer cell lines and one non-cancerous cell line. Remarkably, these complexes exhibited higher toxicity against cancer cells than against the non-cancerous cell line, and they showed greater selectivity than carboplatin, a commonly used chemotherapy drug. Although, in general, these complexes did not surpass the selectivity of gemcitabine, complex **2c** stood out for exhibiting a selectivity index value similar to that of gemcitabine against melanoma cells. Among the series, compounds **2a**–**2c** demonstrated the highest activity, with **2a** being the only complex with some selective activity against lung cancer. Complex **2b** was the most active, though with low selectivity, while complex **2c** exhibited the highest selectivity for melanoma and bladder cancer (selectivity index of 3.0).

## 1. Introduction

The development of transition metal complexes with anticancer potential continues to be a dynamic field of research [1,2,3,4]. A key goal in this area is to identify compounds with low toxicity toward non-cancerous cells, making metals such as copper [5] and zinc [6,7] particularly attractive for study due to their relatively low toxicity. Zinc, in particular, is an essential trace element in the human body because it is a part of numerous enzymes [8,9] and plays an important role in several biological processes [10,11]. The coordination chemistry of Zn^2+^ is rich and versatile, with a wide range of well-characterized complexes involving O-, N- and S-donor ligands [12]. Furthermore, its d^10^ configuration supports various stable coordination geometries—such as tetrahedral, trigonal bipyramidal, and octahedral—and, in general, the resulting complexes are susceptible to easy ligand exchange [13].

Previously, we synthesized and characterized zinc complexes with imidazolium-based dicarboxylate amino acid-derived ligands [14], as well as related silver derivatives that exhibited antimicrobial activity [15]. Both systems showed water solubility and biocompatibility [14], making them promising candidates for further investigation in anticancer applications. Here, we expand this series to include new zinc complexes: [Zn(L^R^)_2_]_n_ (R = Gly, **2a**, and βAla, **2b**), [Zn(L^Leu^)_2_(H_2_O)_2_]_n_ (**2c**), and [Zn(L^R^)(aa)(H_2_O)]_n_ (R = aa = Val, **2d**, and R = aa = Ile, **2e**). Continuing our interest in amino acid-derived transition metal complexes [15,16,17,18] and their applications as antimicrobial [19,20] and anticancer agents [21], we now report on the anticancer activity of these zinc complexes (**2a**–**2e**) against three human cancer cell lines: melanoma cells (MeWo), lung adenocarcinoma (A549), and bladder cancer cells (T24). These cancers are among the most common in the world and have high mortality rates in advanced stages [22]. Lung cancer remains the leading cause of cancer-related mortality worldwide [22], with approximately 40% of patients diagnosed at the metastatic stage, where the five-year survival rate is below 10%. Bladder cancer, although associated with high survival rates in the early stages, has a dramatically reduced five-year survival rate of less than 9% once metastasized. Melanoma, while often treatable in the early stages, also shows a substantial drop in prognosis at the metastatic stage, with a five-year survival rate of approximately 35% [23]. Cytotoxic activity was evaluated in comparison with that of a human non-malignant cell line (skin cells, HaCaT) to determine therapeutic selectivity. This cell line, derived from adult tissue, is characterized by a rapid rate of cell proliferation [24]. Most cancer patients experience side effects during treatment, often necessitating dose reductions or even discontinuation. These side effects arise because most current cancer drugs target rapidly dividing cells, affecting both cancer cells and healthy cells.

## 2. Results

### 2.1. Syntheses and Characterization of Complexes **2a**–**2e**

**Scheme 1:**
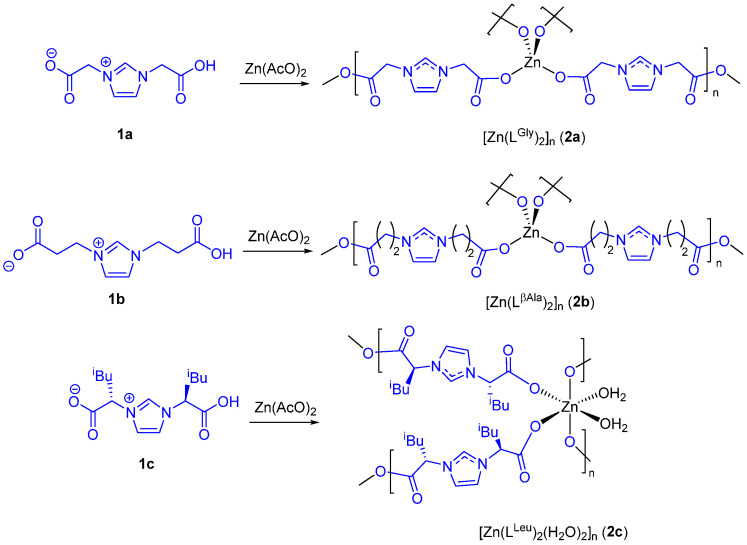
Synthesis of complexes **2a**–**2c** (in EtOH/H_2_O at 70 °C for 1 h).

The reaction of zinc acetate with HL^R^ compounds, **1**, produced complexes [Zn(L^R^)_2_] (R = Gly, **2a**; βAla, **2b**; Leu, **2c**). The isotropic nature of the Zn(II) d^10^ configuration allowed the isolation of these compounds in the solid state as [Zn(L^R^)_2_]_n_ (**2a** and **2b**) or [Zn(L^Leu^)_2_(H_2_O)_2_]_n_ (**2c**) polymeric complexes, featuring four-and six-coordinated zinc environments, respectively (Scheme 1). Complexes **2a**–**2c** were obtained as air-stable crystalline white solids that are water-soluble but sparingly soluble in organic solvents. Infrared (FTIR) spectroscopy showed broad absorptions for the antisymmetric COO stretching vibrations of the carboxylate groups in the 1655–1600 cm^−1^ range, indicating coordination of the [L^R^]^−^ ligands to zinc (Figure S1). This coordination was evidenced by the shift to lower wavenumbers in comparison to those of the HL^R^ ligand precursors, **1**. Symmetric COO vibrations were observed in the 1375–1350 cm^−1^ range, and the difference values of (νCOO_asym_-νCOO_sym_) suggested the κ^1^-O coordination of the carboxylate group [25], which was consistent with the structural characterization discussed later. Similar FTIR absorption patterns have been reported for analogous zinc derivatives [16,26,27]. The ^1^H and ^13^C{^1^H} NMR spectra of **2a**–**2c** (in deuterated water; see Figure S1) displayed signals corresponding to the [L^R^]^−^ ligands, with chemical shifts different from those of the HL^R^ precursors, **1**, due to zinc coordination [28,29]. The spectroscopic properties of complex **2a** are similar to those reported in the bibliography [26,30,31].

**Scheme 2:**
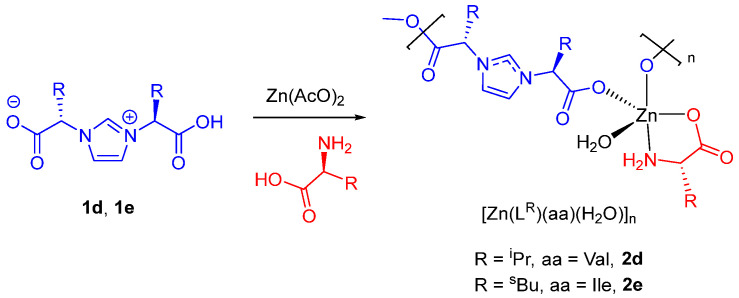
Synthesis of complexes **2d** and **2e** (in EtOH/H_2_O at 70 °C for 1 h).

When zinc acetate was reacted with HL^R^, **1**, in the presence of one equivalent of an amino acid, L-valine or L-isoleucine, complexes **2d** and **2e** were obtained, respectively (Scheme 2). They were identified in the solid state as polymeric complexes [Zn(L^R^)(aa)(H_2_O)]_n_ (R = aa = Val, **2d**, and R = aa = Ile, **2e**). Complexes **2d** and **2e** were also obtained as air-stable water-soluble crystalline white solids that are sparingly soluble in organic solvents. Their FTIR spectra showed carboxylate stretching vibrations, again consistent with κ^1^-O coordination (Figure S1), confirmed by the structural characterization of **2d** and **2e** (see below). The ^1^H and ^13^C{^1^H} NMR spectra of these complexes, in deuterated water, included signals for both the [L^R^]^−^ ligands and the coordinated amino acid (Figure S1). For example, the *sec*-butyl group of the coordinated amino acid Ile in **2e** was clearly identified by its characteristic signals observed in the NMR spectra (assignments detailed in the Section 3 and Figure S1).

### 2.2. Structural Characterization of Complexes **2a**–**2e**

**Figure 1:**
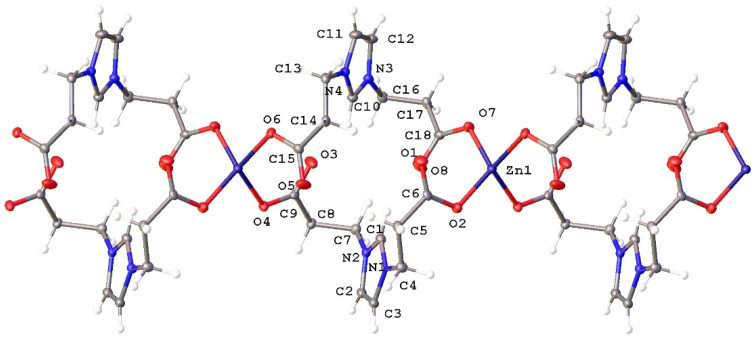
1D coordination polymer of complex **2b** aligned along the *b* axis with ellipsoids of the ORTEP representation displayed with 50% probability.

The identification of complexes **2** as coordination polymers in the solid state was confirmed using X-ray crystallography. The structure of complex **2a** was similar to that previously described [30,31] and is not discussed in detail here (see Figure S6 and Tables S1 and S2 in the Supplementary Materials). The compound [Zn(L^βAla^)_2_]_n_, **2b**, crystallizes in the monoclinic *C***_C_** space group, forming a one-dimensional (1D) coordination polymer that extends along the *b* axis (Figure 1). Zinc ions are linked by bridging [L^βAla^]^−^ anions through carboxylate functionalities that adopt a μ-κ^1^-O^1^,κ^1^-O^3^ coordination mode. This results in asymmetric C-O bond lengths within carboxylate groups (e.g., C(6)-O(1), 1.219(7) and C(6)-O(2), 1.268(7) Å). Other selected structural parameters are listed in Table S2. The zinc ion exhibits a tetrahedral coordination geometry, as occurred in related derivatives [Zn(L^R^)_2_]_n_ (R = ^i^Pr, CH_2_Ph) [16], with a four-coordinate geometry index of *τ*_4_ = 0.85. Non-coordinated C = O groups in carboxylates form weak non-classical C = O^…^H-C hydrogen bonds with adjacent 1D chains (Figure S2), resulting in the observed three-dimensional (3D) crystal packing arrangement (Figure S3).

**Figure 2:**
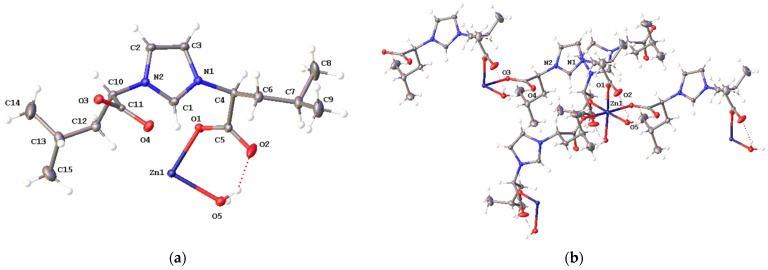
(**a**) ORTEP representation of the asymmetric unit of **2c,** with ellipsoids displayed with 50% probability. (**b**) Polymeric **2c** growth showing the octahedral coordination of zinc.

Complex **2c** crystallizes in the trigonal space group *P*3**_2_**21, forming a two-dimensional (2D) metal–organic framework where zinc ions are interconnected by bridging [L^Leu^]^−^ anions through carboxylate functionalities. The asymmetric unit of **2c** consists of a Zn^2+^ ion, a [L^Leu^]^−^ ligand, and one water molecule (Figure 2a). An intramolecular hydrogen bond is observed between the water ligand and one oxygen atom of the carboxylate (O(5)^…^O(2) distance of 2.649(3) Å). The zinc center adopts a distorted octahedral coordination geometry, surrounded by six oxygen atoms from [L^Leu^]^−^, water ligands, and their symmetry-related counterparts (Figure 2b). Each carboxylate group is coordinated to zinc in a monodentate mode, μ-κ^1^-O^1^,κ^1^-O^3^, leading to asymmetric C-O distances (e.g., C(5)-O(1), 1.254(3) and C(5)-O(2), 1.237(3) Å). The asymmetry in the second carboxylate group is less pronounced (C(11)-O(3), 1.252(3) and C(11)-O(4), 1.248(3) Å) because the O(4) atom participates in an intermolecular hydrogen bond with the water ligand (O(5)^…^O(4)#1 distance of 2.683(3) Å). The other selected structural parameters are summarized in Table S2. The *pseudo-trans* arrangement of the carboxylate groups in the [L^Leu^]^−^ ligand (torsion angle C_carboxy_–C_chiral_–C′_chiral_–C′_carboxy_ of approximately 106°) facilitates a square lattice topology (sql, Figure S4c), creating a 2D distribution of lamellar sheets (Figure S4a,b).

**Figure 3:**
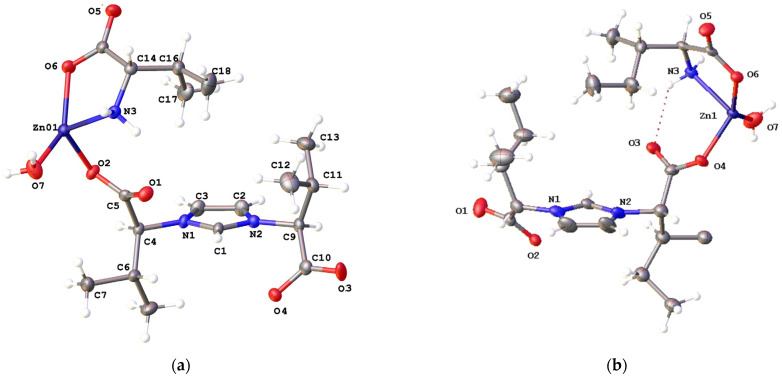
ORTEP representation of asymmetric units of **2d** (**a**) and **2e** (**b**), with ellipsoids displayed with 50% probability.

Complexes **2d** and **2e** crystallize in the monoclinic space groups *C*_2_ and *P*2_1_, respectively. The asymmetric units of **2d** and **2e** contain a Zn(II) ion, the imidazolium dicarboxylate ligand ([L^Val^]^−^ for **2d** and [L^Ile^]^−^ for **2e**), a bidentate amino acid ligand (valine for **2d** and isoleucine for **2e**), and one water molecule (Figure 3). An intramolecular hydrogen bond is observed within this unit between the coordinated amino acid NH_2_ group and one oxygen atom of the carboxylate group of [L^R^]^−^ (N(3)…O(1) distance of 2.962(4) Å for **2d** and N(3)…O(3) distance of 2.993(11) Å for **2e**). The growth of the asymmetric units forms 1D polymeric structures (Figure S5a for **2d**) in which the bridging ligands, [L^Val^]^−^ and [L^Ile^]^−^, coordinate to zinc ions in a monodentate mode, μ-κ^1^-O^1^,κ^1^-O^3^, as occurred in **2a**–**2c**. In both complexes, the zinc center exhibits a bipyramidal trigonal geometry in which the equatorial positions are occupied by three oxygen donor atoms of water, the carboxylate of isoleucine, and one of the carboxylate groups of the [L^Val^]^−^ or [L^Ile^]^−^ ligand. In **2d**, the asymmetry of the three carboxylate C-O distances is clearly observed, while in **2e**, the asymmetry is more pronounced in the isoleucine ligand (C(16)-O(5), 1.239(13) and C(16)-O(6), 1.264(13) Å) compared to the [L^Ile^]^−^ ligand. Other selected structural parameters are detailed in Table S2. The 3D packing occurs through intermolecular hydrogen bonds between the water ligand and one oxygen atom of the carboxylate group (for **2d**: O(7)^…^O(5)#1 and O(7)^…^O(3)#2 distances of 2.610(4) and 2.727(4) Å, respectively; Figure S5b).

### 2.3. Evaluation of Anticancer Activity

**Figure 4:**
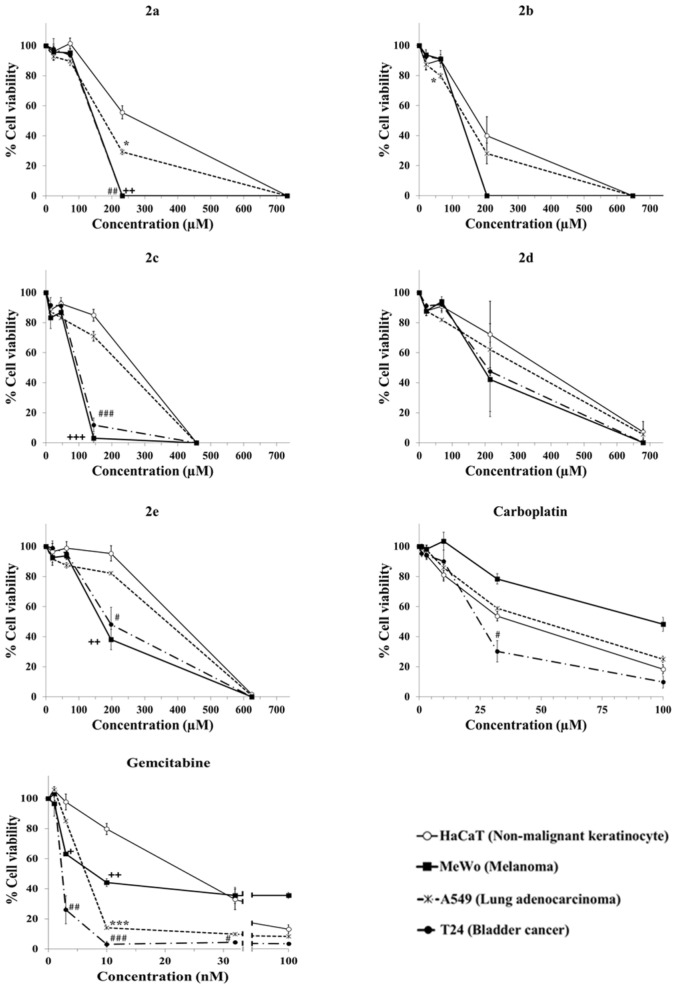
Effect of complexes **2**, carboplatin and gemcitabine, on the viability of human non-malignant cells (HaCaT) and human cancer cells (A549, MeWo, and T24). The cells were exposed to reagents for 72 h, and cell viability was measured using the resazurin assay. Data represent mean ± SEM from at least three independent experiments. For statistical analysis, the *t*-test (paired, two-tailed) was used. * *p* < 0.05, ** *p* < 0.01, and *** *p* <0.001 (similar representations for marks + or #).

The cytotoxicity of complexes **2** was evaluated in vitro by determining the half inhibitory concentrations (IC_50_) against four human cell lines: wild-type BRAF melanoma (MeWo), lung adenocarcinoma (A549), bladder cancer (T24), and non-cancerous skin keratinocytes (HaCaT). The IC_50_ values were calculated from cell viability values obtained after 72 h of exposure of the drug to cells using the resazurin assay. The viability results, summarized in Table 1 (as μM concentrations) and illustrated in Figure 4, also include the values of the selectivity index (SI). The SI was calculated by dividing the IC_50_ value in the non-cancerous cells by that in the cancer cells [32]. The higher the SI value, the higher the selectivity of the compound against cancer cells. This parameter also allows for a comparison of the selectivity of the investigated compound with the selectivity of the standard anticancer drug. For comparison, the cytotoxicity of two clinical anticancer drugs, carboplatin and gemcitabine, as well as imidazolium dicarboxylate precursor ligands (HL^R^, **1**) was evaluated under identical experimental conditions (see viability graphs in Figure 4 and Figure S7). Complexes **2** demonstrated cytotoxic activity against the three cancer cell lines, with IC_50_ values ranging from 75 to 312 μM (Table 1), while the HL^R^ precursor ligands, **1**, did not show cytotoxicity (IC_50_ > 3000 μM, see Figure S7). Two noteworthy observations can be drawn from Table 1. First, although complexes **2** exhibited lower selectivity compared to gemcitabine, they showed greater selectivity than carboplatin, a well-established clinical anticancer drug. Carboplatin is used for the treatment of ovarian cancer, lung cancer, bladder cancer, and head and neck cancer. Second, these zinc-based complexes were more cytotoxic to cancer cells than to non-malignant HaCaT cells, underscoring their potential therapeutic selectivity.

For lung adenocarcinoma (A549), **2a** demonstrated moderate activity with some selectivity, while the remaining complexes showed limited efficacy. The IC_50_ values for **2a**–**2c** are comparable to those reported for a telmisartan zinc derivative that contains only O-donor ligands (IC_50_ 75 μM) [33]. In particular, other zinc complexes with N- or S-donor ligands show lower IC_50_ values ≤ 10 μM) [7], suggesting a ligand-dependent effect [34,35,36]. In the case of T24 bladder cancer cells, complexes **2a**–**2c** exhibited IC_50_ values between 83 and 124 μM (Table 1), with **2c** showing the highest selectivity (close to 3). These results are consistent with related zinc complexes [37]. A particularly strong IC_50_ for T24 cells was previously reported for an oxoaporphine–zinc complex, although the free ligand itself also showed significant cytotoxicity (IC_50_ 9.12 μM) [38]. In melanoma cells (MeWo), complexes **2a**–**2c** achieved the best IC_50_ values (~75 μM for **2c**, Table 1), but no comparable studies were identified in the literature involving this melanoma subtype [6,7]. Importantly, the selectivity of **2c** against this cancer was high, surpassing carboplatin and approaching that of gemcitabine (see Figure S8). Interestingly, the viability of melanoma cells exposed to 145 μM of **2c** was reduced to less than 3%, while the viability of non-malignant cells remained high (~85%). On the contrary, even at the highest concentrations of gemcitabine tested, the cell viability of melanoma cells did not drop below 35%, while the viability of non-malignant cells decreased to 13% (Figure 4). The reduced anticancer activity of the **2d**–**2e** complexes, compared to **2a**–**2c**, can be attributed to the bidentate coordination of the amino acid ligand. Zinc release through dissociation would be more hindered due to the chelate effect than observed in complexes **2a**–**2c**, which have only monodentate ligands. Previous studies suggested that cytotoxicity in zinc complexes, with a reduction in levels of the mutp53 protein, was associated with the release of zinc ions by dissociation [39]. The presence of chelating amino acid ligands in **2d**–**2e** hinders this dissociation, thus reducing its efficacy. Our results on the anticancer activity of these compounds are preliminary and require in vivo studies to confirm their anticancer effects and reveal potential toxicities in normal cell types beyond keratinocytes. Nonetheless, the selectivity indices observed in our panel of cell lines are higher than those of several commonly used anticancer drugs (see Table S3), supporting the relevance of our in vitro findings.

## 3. Materials and Methods

### 3.1. General

All synthetic preparations and other operations were carried out under aerobic conditions. Solvents were purified and dried appropriately prior to use, using standard procedures. Cell culture reagents were purchased from Biowest (Nuaillé, France). Resazurin was purchased from Sigma, gemcitabine was obtained from Pfizer, and carboplatin was obtained from Teva. Other chemicals were obtained from commercial sources and used as supplied. Infrared spectra were recorded on a PerkinElmer FT-IR Spectrum Two spectrophotometer (Waltham, MA, USA) using the ATR technique. NMR spectra were recorded on Bruker AMX-300 or Avance III spectrometers (Billerica, MA, USA) at the Centro de Investigaciones, Tecnología e Innovación (CITIUS) of the University of Sevilla, with ^1^H and ^13^C{^1^H} NMR shifts referenced to residual signals from deuterated solvents. All data are reported in ppm downfield from Si(CH_3_)_4_. Elemental analyses (C, H, N) were conducted by the CITIUS of the University of Sevilla on an Elemental LECO CHNS 93 analyzer (LECO Corporation, St. Joseph, MI, USA). Bis-imidazolium precursors were prepared according to the literature experimental methods [28,29,40]. Complex **2a** was prepared with slight differences from the procedure reported [30].

### 3.2. Synthesis

#### 3.2.1. Complexes [Zn(L^R^)_2_]_n_, **2a** and **2b**

A solution of Zn(AcO)_2_·2H_2_O (0.110 g, 0.5 mmol) in 10 mL of ethanol was added to HL^Gly^ (0.184 g, 1 mmol) dissolved in the smallest amount of a 3:1 ethanol–water mixture. The resulting solution was stirred for 1 h at 70 °C. The solvent was then removed under reduced pressure and an oil was obtained, which was recrystallized in a 1:1 dimethylformamide–water mixture. The compound [Zn(L^Gly^)_2_]_n_, **2a**, was obtained as an off-white solid (0.59 g, 47 %). IR (ATR, cm^−1^): 3140 (w), 3081 (w), 3003 (w), 2876 (w), 2168 (w), 1652 (vs, ν_as_ COO), 1562 (s), 1433 (m), 1416 (m), 1379 (vs), 1362 (vs), 1350 (vs, ν_s_ COO), 1323 (m), 1290 (vs), 1207 (w), 1188 (m), 1167 (s), 1109 (m), 1035 (m), 976 (w), 870 (m), 802 (s), 674 (vs), 636 (s), 585 (vs), 496 (s), 442 (m), 431 (m). ^1^H NMR (300 MHz, D_2_O): 8.68 (br t, 2H, C*H*_im_), 7.38 (d, ^3^J_HH_ = 2 Hz, 4H, C*H*_im_), 4.77 (s, 8H, C*H*_2_COO). ^13^C{^1^H} NMR (D_2_O, 75 MHz): 172.3 (*C*OO), 137.2 (N-*C*H_im_-N), 123.2 (*C*H_im_), 51.9 (*C*H_2_COO). Elemental Anal. Calc. for C_17_H_21_N_5_O_9_Zn (**2a** + DMF): C, 40.45; H, 4.19; N, 13.87. Found: C, 39.68; H, 3.48; N, 13.52%. 

Complex [Zn(L^βAla^)_2_]_n_, **2b**, was prepared following the same method, using HL^βAla^ (212 mg, 1 mmol) and Zn(AcO)_2_·2H_2_O (110 mg, 0.5 mmol); **2b** was obtained as a pale white solid (0.096 g, 39 %). IR (ATR, cm^−1^): 3383 (m), 3142 (m), 3108 (m), 3007 (w), 2965 (w), 1604 (vs, ν_as_ COO), 1569 (vs), 1559 (vs), 1450 (m), 1395 (vs), 1372 (vs, ν_s_ COO), 1325 (s), 1300 (s), 1244 (m), 1229 (m), 1182 (s), 1150 (vs), 1100 (m), 1066 (m), 1023 (w), 988 (w), 943 (m), 864 (m), 825 (m), 775 (m), 755 (m), 650 (s), 634 (s), 604 (s), 559 (m), 532 (m), 519 (m), 434 (w), 418 (w). ^1^H NMR (300 MHz, D_2_O): 8.70 (br t, 2H, C*H*_im_), 7.44 (d, ^3^J_HH_ = 2 Hz, 4H, C*H*_im_), 4.36 (t, ^3^J_HH_ = 7 Hz, 8H, C*H*_2_CH_2_COO), 2.72 (t, ^3^J_HH_ = 7 Hz, 8H, CH_2_C*H*_2_COO). ^13^C{^1^H} NMR (D_2_O, 75 MHz): 178.1 (*C*OO), 136.0 (N-*C*H_im_-N), 122.2 (*C*H_im_), 46.7 (*C*H_2_CH_2_COO), 37.1 (CH_2_*C*H_2_COO). Elemental Anal. Calc. for C_18_H_22_N_4_O_8_Zn (**2b**): C, 44.32; H, 4.55; N, 11.49. Found: C, 44.17; H, 4.65; N, 11.31%. 

#### 3.2.2. Complex [Zn(L^Leu^)_2_(H_2_O)_2_]_n_, **2c**

The experimental method was similar to **2a**,**b**, using HL^Leu^ (296 mg, 1 mmol) and Zn(AcO)_2_·2H_2_O (109 mg, 0.5 mmol). From crystallization in a DMF:H_2_O 1:1 mixture **2c** was obtained as a pale white solid with the formula [Zn(L^Leu^)_2_(H_2_O)_2_]_n_ (0.165 g, 47 %). IR (ATR, cm^−1^): 3143 (w), 2956 (m), 2871 (w), 1621 (vs, ν_as_ COO), 1467 (w), 1435 (w), 1372 (vs, ν_s_ COO), 1284 (w), 1234 (w), 1156 (m), 900 (w), 827 (w), 738 (w), 698 (m), 651 (m), 618 (w), 501 (ws), 418 (w). ^1^H NMR (300 MHz, D_2_O): 8.93 (br t, 1H, C*H*_im_), 7.50 (d, ^3^J_HH_ = 1.3 Hz, 2H, C*H*_im_), 4.86 (dd, ^3^J_HH_ = 10.2, 5.5 Hz, 2H, C*H*COO), 1.95 (m, 4H, (CH_3_)_2_CHC*H_2_*), 1.24 (hp, ^3^J_HH_ = 6.6 Hz, 2H, (CH_3_)_2_C*H*CH_2_), 0.82 (d, ^3^J_HH_ = 6.6 Hz, 3H, (C*H_3_*)_2_CHCH_2_), 0.81 (d, ^3^J_HH_ = 6.6 Hz, 3H, (C*H_3_*)_2_CHCH_2_CH). ^13^C{^1^H} NMR (D_2_O, 75 MHz): 174.9 (*C*OO), 135.4 (N-*C*H_im_-N), 121.7 (*C*H_im_), 63.57 (*C*HCOO), 40.67 ((CH_3_)_2_CH*C*H_2_), 24.5 ((CH_3_)_2_*C*HCH_2_), 21.99 ((*C*H_3_)_2_CHCH_2_), 20.29 ((*C*H_3_)_2_CHCH_2_). Elemental Anal. Calc. for C_30_H_50_N_4_O_10_Zn (**2c**): C, 52.06; H, 7.28; N, 8.10. Found: C, 54.94; H, 7.29; N, 8.05%. 

#### 3.2.3. Complexes [Zn(L^R^)(aa)(H_2_O)]_n_, **2d** and **2e**

[Zn(L^Val^)(Val)(H_2_O)]_n_, **2d**.

Following the same method as in **2a**, using the precursor HL^Val^ (134 mg, 0.5 mmol) and Zn(AcO)_2_·2H_2_O (110 mg, 0.5 mmol) in the presence of L-valine (58 mg, 0.5 mmol). Complex **2d** was obtained as a pale white solid after crystallization from a DMF:H_2_O 3:1 mixture. Yield: 0.135 g (56%). IR (ATR, cm^−1^): 3296 (w), 3260 (w), 3138 (w), 2970 (w), 2873 (w), 1607 (vs, ν_as_ COO), 1586 (vs), 1466 (m), 1414 (m), 1375 (vs, ν_s_ COO), 1330 (m), 1306 (m), 1263 (w), 1236 (m), 1181 (w), 1156 (m), 1121 (w), 1095 (m), 1064 (m), 1015 (w), 987 (w), 950 (w), 916 (w), 863 (w), 847 (w), 816 (w), 789 (w), 773 (m), 749 (s), 730 (m), 703 (s), 665 (m), 635 (s), 614 (s), 585 (m), 543 (w), 502 (m), 453 (w), 423 (w), 409 (w). ^1^H NMR (300 MHz, D_2_O): 8.90 (t, 1H, ^3^J_HH_ = 1.8 Hz, C*H*_im_), 7.52 (d, ^3^J_HH_ = 1.8 Hz, 2H, C*H*_im_), 4.53 (d, ^3^J_HH_ = 8.1 Hz, 2H, NC*H*COO), 3.40 (br d, 1H, ^3^J_HH_ = 3 Hz, NC*H*COO Val), 2.39 (hp, 2H, ^3^J_HH_ = 6.9 Hz, C*H*(CH_3_)_2_), 2.26 (m, 1H, C*H*(CH_3_)_2_ Val), 0.96–0.90 (m, 9H, CH(C*H*_3_)_2_), 0.84–0.79 (m, 9H, CH(C*H*_3_)_2_). ^13^C{^1^H} NMR (D_2_O, 75 MHz): 173.8 (*C*OO), 135.7 (N-*C*H_im_-N), 122.0 (*C*H_im_), 71.5 (N*C*HCOO), 59.5 (N*C*HCOO Val), 31.1 (*C*H(CH_3_)), 29.5 (*C*H(CH_3_) Val), 18.6 (CH(*C*H_3_)), 18.4 (CH(*C*H_3_) Val), 17.5 (CH(*C*H_3_)), 15.8 (CH(*C*H_3_) Val). Elemental Anal. Calc. for C_18_H_31_N_3_O_7_Zn (**2d**): C, 46.31; H, 6.69; N, 9.00. Found: C, 46.70; H, 6.53; N, 8.81%.

[Zn(L^Ile^)(Ile)(H_2_O)]_n_, **2e**.

Following the same method as in **2a**, using the HL^Ile^ precursor (149 mg, 0.5 mmol) and Zn(AcO)_2_·2H_2_O (110 mg, 0.5 mmol) in the presence of L-isoleucine (66 mg, 0.5 mmol). Complex **2e** was obtained as a pale white solid after crystallization from a DMF:H_2_O 3:1 mixture. Yield: 0.11 g (42%). IR (ATR, cm^−1^): 3136 (w), 2968 (m), 2933 (w), 2881 (w), 1631 (vs, ν_as_ COO), 1615 (vs), 1584 (vs), 1512 (s), 1460 (m), 1421 (w), 1363 (vs, ν_s_ COO), 1329 (s), 1307 (m), 1256 (m), 1234 (w), 1155 (m), 1099 (w), 1062 (w), 1026 (w), 961 (w), 919 (w), 870 (w), 840 (w), 746 (s), 709 (m), 649 (m), 615 (m), 559 (s), 537 (s), 455 (s). ^1^H NMR (300 MHz, D_2_O): 8.89 (t, ^3^J_HH_ = 2 Hz, 1H, C*H*_im_), 7.51 (d, ^3^J_HH_ = 2 Hz, 2H, C*H*_im_), 4.56 (d, ^3^J_HH_ = 8.1 Hz, 2H, NC*H*COO), 3.42 (br, 1H, NC*H*COO Ile), 2.17 (m, 2H, CH_3_C*H*CH_2_CH_3_), 1.95 (m, 1H, CH_3_C*H*CH_2_CH_3_ Ile), 1.25–1.11 (m, 6H, CH_3_CHC*H*_2_CH_3_), 0.97-0.88 (m, 9H, C*H*_3_CHCH_2_CH_3_), 0.82-0.76 (m, 9H, CH_3_CHCH_2_C*H*_3_). ^13^C{^1^H}-NMR (D_2_O, 75 MHz): 173.9 (*C*OO), 135.6 (N-*C*H_im_-N), 122.0 (*C*H_im_), 70.5 (N*C*HCOO), 59.1 (N*C*HCOO Ile), 36.9 (CH_3_*C*HCH_2_CH_3_), 36.5 (CH_3_*C*HCH_2_CH_3_ Ile), 24.6 (CH_3_CH*C*H_2_CH_3_), 23.8 (CH_3_CH*C*H_2_CH_3_ Ile), 15.2 (*C*H_3_CHCH_2_CH_3_ Ile), 15.1 (*C*H_3_CHCH_2_CH_3_), 11.2 (CH_3_CHCH_2_*C*H_3_ Ile), 10.3 (CH_3_CHCH_2_*C*H_3_). Elemental Anal. Calc. for C_21_H_34_N_3_O_7_Zn (**2e**): C, 49.86; H, 6.77; N, 8.31. Found: C, 51.23; H, 7.50; N, 8.46%. 

### 3.3. Cell Lines and Cell Viability Assays

HaCaT cells (human keratinocytes) [24] A549 (human lung adenocarcinoma), MeWo (human melanoma), and T24 (human urinary bladder carcinoma) were purchased from Cell Lines Service (CLS). The cells were maintained in Dulbecco’s Modified Eagle Medium (DMEM) supplemented with 10% fetal bovine serum and 1% penicillin/streptomycin. All cell lines were cultured at 37 °C and 5% CO_2_ with saturating humidity.

The resazurin assay is a commonly used colorimetric/fluorescent method to assess cell viability. This approach is based on the ability of living cells to convert the blue dye resazurin into the pink soluble resorufin product. The number of viable cells is directly correlated with the amount of resorufin generated. Between 3000 and 6000 cells per well were seeded in 96-well plates and allowed to grow for 24 h. The cells were then exposed to several concentrations of test compounds or anticancer drugs carboplatin and gemcitabine for 72 h. The compounds were dissolved in sterile water to prepare a stock solution. Working solutions were obtained by serial dilution in the culture medium and were used immediately to treat the cells. Following this treatment period, the cells were rinsed once with phosphate buffered saline (PBS), and 150 μL of resazurin solution (20 µg/mL in medium) was added to each well. The plates were then incubated for 4–5 h at 37 °C with 5% CO_2_. Finally, absorbance was measured at 540 and 620 nm using a multiwell plate spectrophotometer (Imark Bio Rad Laboratories Inc., Hercules, CA, USA). The results were reported as percentages of cell viability compared to untreated controls. All data represent the mean ± standard error of the mean (SEM) of at least three independent experiments. The selectivity index (SI) values for each cancer cell line were determined by calculating the ratio of the IC_50_ value in the non-malignant HaCaT cell line to the IC_50_ value in the corresponding cancer cell line, based on data from each independent experiment.

A paired two-tailed *t*-test was performed for statistical analysis. We compared the cytotoxicity of a particular concentration of the compound between HaCaT and A549 (asterisks, *) or HaCaT and MeWo (cross, +) or HaCaT and T24 (hashes, #). A *p* value > 0.05 is not considered statistically significant and was not marked. A *p* value < 0.05 is considered statistically significant and is marked by the symbols * or + or #; a *p* value < 0.01 by ** or ++ or ##; and a *p* value < 0.001 by *** or +++ or ### (Figure 4).

### 3.4. Single-Crystal X-Ray Crystallography

A summary of the crystallographic data and the structure refinement results for compounds **2a**–**2e** is given in Table S1. Crystals of a suitable size for X-ray diffraction analysis were coated with dry perfluoropolyether, mounted on glass fibers, and fixed in a cold nitrogen stream (T = 193 K) to the goniometer head. Data collection was carried out on a Bruker-AXS, D8 QUEST ECO, PHOTON II area detector diffractometer using monochromatic radiation *λ*(Mo K_α_) = 0.71073 Å, by means of ω and φ scans with a width of 0.50 degrees. The data were reduced (SAINT [41]) and corrected for absorption effects using the multi-scan method (SADABS [42]). The structure was solved using direct methods (SIR-2002 [43]) and refined against all *F*^2^ data using full-matrix least-squares techniques (SHELXTL-2018/3 [44]) minimizing *w*[*F*_o_^2^ − *F*_c_^2^]^2^. All non-hydrogen atoms were refined anisotropically. The hydrogen atoms were included from the calculated positions and refined riding on their respective carbon atoms with isotropic displacement parameters. The **2d** crystal structure was refined as a two-component inversion twin in a refinement ratio of 0.891:0.109. A search for solvent-accessible voids for the crystal structure of **2e** using SQUEEZE [45] revealed two identical voids, each with a volume of 246 Å^3^ and an electron count of 77. Although the solvent content could not be definitively identified or refined under the most stringent constraints, the observed volume and electron density are consistent with the presence of one DMF molecule and three disordered water molecules in each void. The corresponding CIF data represent SQUEEZE-treated structures with solvent molecules handling as a diffuse contribution to the overall scattering, without specific atom position and excluded from the structural model. The SQUEEZE results were appended to the CIF. The corresponding crystallographic data were deposited with the Cambridge Crystallographic Data Centre as supplementary publications. CCDC 2,411,227 (**2a**), 2,411,228 (**2b**), 2,411,229 (**2c**), 2,411,230 (**2d**), and 2,411,231 (**2e**) contain the supplementary crystallographic data for this paper. The data can be obtained free of charge via https://www.ccdc.cam.ac.uk/structures/ (accessed on 28 March 2025). 

## 4. Conclusions

The complexes [Zn(L^R^)_2_]_n_ (R = Gly, **2a**, βAla, **2b**), [Zn(L^Leu^)_2_(H_2_O)_2_]_n_, **2c**, and [Zn(L^R^)(aa)(H_2_O)]_n_ (R = aa = Val, **2d**, Ile, **2e**) were synthesized by reacting the appropria5e imidazolium-dicarboxylate compound HL^R^, **1**, with zinc acetate, under appropriate reaction conditions. They were characterized spectroscopically and structurally identified as coordination polymers in which imidazolium-dicarboxylates [L^R^]^−^ acted as bridging monodentate ligands in a μ-κ^1^-O^1^,κ^1^-O^3^ coordination mode. Diverse coordination geometries for zinc(II) were observed in these complexes: tetrahedral for **2a** and **2b**, octahedral for **2c**, and trigonal bipyramidal for **2d** and **2e**. The cytotoxic behavior of complexes **2a**–**2e** was evaluated against four human cell lines: lung adenocarcinoma (A549), melanoma BRAF WT (MeWo), bladder cancer (T24), and a non-malignant skin cell line (HaCaT). These Zn-based complexes demonstrated two notable features with respect to their potential therapeutic applications. First, they exhibited greater selectivity, in general, compared to carboplatin, a well-stablished chemotherapy drug used to treat various types of cancer. Second, they were more cytotoxic to cancer cell lines than to non-malignant HaCaT cells. Among the compounds tested, complexes **2a**–**2c** showed the highest anticancer activity than the complexes containing coordinated amino acids (**2d** and **2e**). However, moderate activity and limited selectivity, except for **2a**, were observed against A549 cells. Complexes **2b** and **2c** exhibited the most potent activity against melanoma BRAF WT (MeWo) and bladder cancer (T24), with **2c** showing the highest selectivity toward these cancer cell lines (selectivity index of ca. 3.0). Interestingly, the selectivity of **2c** against melanoma surpassed that of carboplatin and approached that of gemcitabine. Ongoing studies aim to enhance the selective anticancer properties of these zinc complexes through the strategic design of ligands.

## Figures and Tables

**Table 1:** IC_50_ values (μM) and selectivity indexes (SIs) of zinc complexes tested against human cell lines ^a^.

Complex	IC_50_ (Mean ± SEM; *p* Value vs. HaCaT) (Selectivity Index, Mean ± SEM)			
	HaCaT (Non-Malignant Keratinocyte)	A549 (Lung Adenocarcinoma)	MeWo (Melanoma)	T24 (Bladder Cancer)
**2a**	260.2 ± 20.0	156.3 ± 3.7; 0.0214 (1.7 ± 0.2)	126.5 ± 1.9; 0.0071 (2.1 ± 0.2)	124.4 ± 5.5; 0.0101 (2.1 ± 0.2)
**2b**	185.0 ± 33.7	131.8 ± 11.2; 0.1048 (1.4 ± 0.1)	108.7 ± 2.2; 0.1139 (1.7 ± 0.3)	108.5 ± 3.9; 0.1183 (1.7 ± 0.3)
**2c**	231.4 ± 7.4	202.4 ± 7.6; 0.0965 (1.1 ± 0.1)	75.7 ± 1.9; 0.0001 (3.1 ± 0.0)	83.2 ± 3.8; 0.0001 (2.8 ± 0.1)
**2d**	328.9 ± 77.8	283.3 ± 59.7; 0.1027 (1.1 ± 0.1)	227.7 ± 66.4; 0.1403 (1.6 ± 0.5)	238.9 ± 74.5; 0.2019 (1.6 ± 0.5)
**2e**	343.9 ± 12.5	311.7 ± 3.2; 0.1299 (1.1 ± 0.1)	162.3 ± 19.3; 0.0032 (2.2 ± 0.3)	194.6 ± 37.7; 0.0157 (1.9 ± 0.3)
Carboplatin	35.9 ± 3.8	42.9 ± 1.4; 0.2188 (0.8 ± 0.1)	100.4 ± 17.4; 0.0182 (0.4 ± 0.0)	22.1 ± 2.6; 0.0130 (1.7 ± 0.2)
Gemcitabine (nM)	22.1 ± 3.1	5.6 ± 0.1; 0.0130 (4.0 ± 0.5)	7.3 ± 0.7; 0.0270 (3.3 ± 0.8)	2.3 ± 0.3; 0.0062 (9.3 ± 0.4)

^a^ After 72 h of treatment, cell viability was measured using the resazurin assay. IC_50_ values are given as the mean value obtained from at least three independent experiments ± standard error of the mean (SEM) (see Section 3 for more details). The selectivity index (SI) values were calculated as the average of the IC_50_ values in the HaCaT non-cancerous cell line divided by the IC_50_ value in the cancer cell line obtained in each independent experiment. For the calculation of the μM concentration, the molecular weight of the monomer of the corresponding coordination polymer was used.

## Data Availability

Data will be made available on request.

## Supplementary Materials

The following supporting information can be downloaded at https://www.mdpi.com/article/10.3390/ijms26073202/s1.

## References

[1] Bruijnincx P.C., Sadler P.J. (2008). New Trends for Metal Complexes with Anticancer Activity. Curr. Opin. Chem. Biol..

[2] Ott I. (2009). On the Medicinal Chemistry of Gold Complexes as Anticancer Drugs. Coord. Chem. Rev..

[3] Ndagi U., Mhlongo N., Soliman M.E. (2017). Metal Complexes in Cancer Therapy—An Update from Drug Design Perspective. Drug Des. Dev. Ther..

[4] Hindi K.M., Panzner M.J., Tessier C.A., Cannon C.L., Youngs W.J. (2009). The Medicinal Applications of Imidazolium Carbene-Metal Complexes. Chem. Rev..

[5] Santini C., Pellei M., Gandin V., Porchia M., Tisato F., Marzano C. (2014). Advances in Copper Complexes as Anticancer Agents. Chem. Rev..

[6] Porchia M., Pellei M., Del Bello F., Santini C. (2020). Zinc Complexes with Nitrogen Donor Ligands as Anticancer Agents. Molecules.

[7] Pellei M., Del Bello F., Porchia M., Santini C. (2021). Zinc Coordination Complexes as Anticancer Agents. Coord. Chem. Rev..

[8] Andreini C., Bertini I. (2012). A Bioinformatics View of Zinc Enzymes. J. Inorg. Biochem..

[9] Lipscomb W.N., Sträter N. (1996). Recent Advances in Zinc Enzymology. Chem. Rev..

[10] Vallee B.L., Falchuk K.H. (1993). The Biochemical Basis of Zinc Physiology. Physiol. Rev..

[11] Krężel A., Maret W. (2016). The Biological Inorganic Chemistry of Zinc Ions. Arch. Biochem. Biophys..

[12] Burgess J., Prince R.H. (2005). Zinc: Inorganic & coordination chemistry. Encyclopedia of Inorganic Chemistry.

[13] Sadow A.D. (2021). 3.06-Zinc. Comprehensive Coordination Chemistry III.

[14] Abendrot M., Chȩcińska L., Kusz J., Lisowska K., Zawadzka K., Felczak A., Kalinowska-Lis U. (2020). Zinc(II) Complexes with Amino Acids for Potential Use in Dermatology: Synthesis, Crystal Structures, and Antibacterial Activity. Molecules.

[15] Caballero P., Colodrero R.M.P., Conejo M.d.M., Pastor A., Álvarez E., Montilla F., Carrasco C.J., Nicasio A.I., Galindo A. (2020). Homochiral Imidazolium-Based Dicarboxylate Compounds: Structure and Solution Behaviour. Inorganica Chim. Acta.

[16] Nicasio A.I., Montilla F., Álvarez E., Colodrero R.P., Galindo A. (2017). Synthesis and Structural Characterization of Homochiral 2D Coordination Polymers of Zinc and Copper with Conformationally Flexible Ditopic Imidazolium-Based Dicarboxylate Ligands. Dalton Trans..

[17] Borrego E., Nicasio A.I., Álvarez E., Montilla F., Córdoba J.M., Galindo A. (2019). Synthesis and Structural Characterization of Homochiral Coordination Polymers with Imidazole-Based Monocarboxylate Ligands. Dalton Trans..

[18] Carrasco C.J., Montilla F., Álvarez E., Conejo M.d.M., Pastor A., Galindo A. (2023). Recent Developments in Amino Acid-Derived Imidazole-, Imidazolium- and N-Heterocyclic Carbene-Carboxylate Complexes. Inorganica Chim. Acta.

[19] Carrasco C.J., Montilla F., Álvarez E., Galindo A., Pérez-Aranda M., Pajuelo E., Alcudia A. (2022). Homochiral Imidazolium-Based Dicarboxylate Silver(I) Compounds: Synthesis, Characterisation and Antimicrobial Properties. Dalton Trans..

[20] Sánchez A., Carrasco C.J., Montilla F., Álvarez E., Galindo A., Pérez-Aranda M., Pajuelo E., Alcudia A. (2022). Antimicrobial Properties of Amino-Acid-Derived N-Heterocyclic Carbene Silver Complexes. Pharmaceutics.

[21] Carrasco C.J., Montilla F., Álvarez E., Calderón-Montaño J.M., López-Lázaro M., Galindo A. (2022). Chirality Influence on the Cytotoxic Properties of Anionic Chiral Bis(N-Heterocyclic Carbene)Silver Complexes. J. Inorg. Biochem..

[22] Bray F., Laversanne M., Sung H., Ferlay J., Siegel R.L., Soerjomataram I., Jemal A. (2024). Global Cancer Statistics 2022: GLOBOCAN Estimates of Incidence and Mortality Worldwide for 36 Cancers in 185 Countries. CA Cancer J. Clin..

[23] Siegel R.L., Kratzer T.B., Giaquinto A.N., Sung H., Jemal A. (2025). Cancer Statistics, 2025. CA Cancer J. Clin..

[24] Boukamp P., Petrussevska R.T., Breitkreutz D., Hornung J., Markham A., Fusenig N.E. (1988). Normal Keratinization in a Spontaneously Immortalized Aneuploid Human Keratinocyte Cell Line. J. Cell Biol..

[25] Sutton C.C.R., Da Silva G., Franks G.V. (2015). Modeling the IR Spectra of Aqueous Metal Carboxylate Complexes: Correlation between Bonding Geometry and Stretching Mode Wavenumber Shifts. Chem.—Eur. J..

[26] Fei Z., Ang W.H., Geldbach T.J., Scopelliti R., Dyson P.J. (2006). Ionic Solid-State Dimers and Polymers Derived from Imidazolium Dicarboxylic Acids. Chem.—Eur. J..

[27] Babu C.N., Sathyanarayana A., Mobin S.M., Prabusankar G. (2013). Structurally Characterized Zwitterionic Chiral Zinc Imidazolium [4,4] Grid. Inorg. Chem. Commun..

[28] Kühl O., Palm G. (2010). Imidazolium Salts from Amino Acids-a New Route to Chiral Zwitterionic Carbene Precursors?. Tetrahedron Asymmetry.

[29] Kühl O., Millinghaus S., Wehage P. (2010). Functionalised, Chiral Imidazolium Compounds from Proteinogenic Amino Acids. Open Chem..

[30] Farger P., Guillot R., Leroux F., Parizel N., Gallart M., Gilliot P., Rogez G., Delahaye E., Rabu P. (2015). Imidazolium Dicarboxylate Based Metal-Organic Frameworks Obtained by Solvo-Ionothermal Reaction. Eur. J. Inorg. Chem..

[31] Fei Z., Zhao D., Geldbach T.J., Scopelliti R., Dyson P.J., Antonijevic S., Bodenhausen G. (2005). A Synthetic Zwitterionic Water Channel: Characterization in the Solid State by X-Ray Crystallography and NMR Spectroscopy. Angew. Chem. Int. Ed..

[32] Lopez-Lazaro M. (2015). A Simple and Reliable Approach for Assessing Anticancer Activity In Vitro. Curr. Med. Chem..

[33] Martínez V.R., Aguirre M.V., Todaro J.S., Ferrer E.G., Williams P.A.M. (2020). Improvement of the Anticancer Activities of Telmisartan by Zn(II) Complexation and Mechanisms of Action. Biol. Trace Elem. Res..

[34] Andres S.A., Bajaj K., Vishnosky N.S., Peterson M.A., Mashuta M.S., Buchanan R.M., Bates P.J., Grapperhaus C.A. (2020). Synthesis, Characterization, and Biological Activity of Hybrid Thiosemicarbazone-Alkylthiocarbamate Metal Complexes. Inorg. Chem..

[35] Naskar B., Modak R., Maiti D.K., Drew M.G.B., Bauzá A., Frontera A., Das Mukhopadhyay C., Mishra S., Das Saha K., Goswami S. (2017). A Schiff Base Platform: Structures, Sensing of Zn(II) and PPi in Aqueous Medium and Anticancer Activity. Dalton Trans..

[36] Tan Y.S., Ooi K.K., Ang K.P., Akim A.M., Cheah Y.K., Halim S.N.A., Seng H.L., Tiekink E.R.T. (2015). Molecular Mechanisms of Apoptosis and Cell Selectivity of Zinc Dithiocarbamates Functionalized with Hydroxyethyl Substituents. J. Inorg. Biochem..

[37] Yu P., Deng J., Cai J., Zhang Z., Zhang J., Hamid Khan M., Liang H., Yang F. (2019). Anticancer and Biological Properties of a Zn-2,6-Diacetylpyridine Bis(Thiosemicarbazone) Complex. Metallomics.

[38] Qin J.L., Shen W.Y., Chen Z.F., Zhao L.F., Qin Q.P., Yu Y.C., Liang H. (2017). Oxoaporphine Metal Complexes (CoII, NiII, ZnII) with High Antitumor Activity by Inducing Mitochondria-Mediated Apoptosis and S-Phase Arrest in HepG2. Sci. Rep..

[39] Xhafa S., Di Nicola C., Tombesi A., Pettinari R., Pettinari C., Scarpelli F., Crispini A., La Deda M., Candreva A., Garufi A. (2024). Pyrazolone-Based Zn(II) Complexes Display Antitumor Effects in Mutant P53-Carrying Cancer Cells. J. Med. Chem..

[40] Carrasco C.J., Montilla F., Villalobo E., Angulo M., Álvarez E., Galindo A. (2024). Antimicrobial Activity of Anionic Bis(N-Heterocyclic Carbene) Silver Complexes. Molecules.

[41] (2007). Bruker SAINT+. SAINT+.

[42] Sheldrick G.M. (1997). SADABS, Programs for Scaling and Absorption Correction of Area Detector Data.

[43] Burla M.C., Camalli M., Carrozzini B., Cascarano G.L., Giacovazzo C., Polidori G., Spagna R. (2003). SIR2002: The Program. J. Appl. Crystallogr..

[44] Sheldrick G.M. (2008). A Short History of SHELX. Acta Crystallogr. Sect. A.

[45] Van Der Sluis P., Spek A.L. (1990). BYPASS: An Effective Method for the Refinement of Crystal Structures Containing Disordered Solvent Regions. Acta Crystallogr. Sect. A.

