# Supplementary material for: Synthesis, Characterization, and Preliminary In Vitro Anticancer Activity of Zinc Complexes Containing Amino Acid-Derived Imidazolium-Based Dicarboxylate Ligands
Source: Int J Mol Sci. 2025 Mar 30;26(7):3202. doi: 10.3390/ijms26073202 (PMC11989707; doi:10.3390/ijms26073202)
Supplement: Supplementary file 1 [file ijms-26-03202-s001.zip › CheckCIF PLATON_report_global_CIF_with_five_structures.pdf]

checkCIF (basic structural check) running

Checking for embedded fcf data in CIF ...
Found embedded fcf data in CIF. Extracting fcf data from uploaded CIF, please wait . . . .

checkCIF/PLATON (basic structural check)

Structure factors have been supplied for datablock(s) 2a, 2b, 2c, 2d, 2e

THIS REPORT IS FOR GUIDANCE ONLY. IF USED AS PART OF A REVIEW PROCEDURE FOR PUBLICATION, IT SHOULD NOT REPLACE THE EXPERTISE OF AN EXPERIENCED CRYSTALLOGRAPHIC REFEREE.

No syntax errors found. CIF dictionary
Please wait while processing .... Interpreting this report

Structure factor report

Datablock: 2a

Bond precision:	C-C = 0.0020 A		Wavelength=0.71073
Cell:	a=17.1975(8)	b=7.4004(3)	c=12.7633(6)
	alpha=90	beta=106.583(2)	gamma=90
Temperature:	193 K		

	Calculated	Reported
Volume	1556.80(12)	1556.80(12)
Space group	C 2/c	C 2/c
Hall group	-C 2yc	-C 2yc
Moiety formula	C14 H14 N4 O8 Zn	C14 H14 N4 O8 Zn
Sum formula	C14 H14 N4 O8 Zn	C14 H14 N4 O8 Zn
Mr	431.68	431.66
Dx,g cm-3	1.842	1.842
Z	4	4
Mu (mm-1)	1.636	1.636
F000	880.0	880.0
F000'	881.57	
h,k,lmax	20,8,15	20,8,15
Nref	1416	1386
Tmin,Tmax	0.822,0.921	0.632,0.746
Tmin'	0.612	

Correction method= # Reported T Limits: Tmin=0.632 Tmax=0.746
AbsCorr = MULTI-SCAN

Data completeness= 0.979	Theta(max)= 25.247	
R(reflections)= 0.0199(1379)		wR2(reflections)= 0.0504(1386)
S = 1.090	Npar= 123	

The following ALERTS were generated. Each ALERT has the format
test-name_ALERT_alert-type_alert-level.
Click on the hyperlinks for more details of the test.

Alert level C

PLAT029_ALERT_3_C _diffrn_measured_fraction_theta_full value Low . 0.979 Why?
PLAT911_ALERT_3_C Missing FCF Refl Between Thmin & STh/L= 0.600 30 Report
0 2 0, 0 6 0, -1 1 1, 1 5 1, -12 0 2, -11 3 2,
-4 2 2, -2 0 2, 1 1 2, 2 0 2, 6 4 2, 0 2 3,
1 5 3, -12 0 4, 1 1 4, 2 0 4, 7 3 4, 6 2 5,
7 1 5, -12 0 6, -11 1 6, -9 3 6, -7 3 6, -10 2 7,
-5 3 8, 2 0 8, -4 2 9, -3 1 9, -10 0 10, -6 0 10,

Alert level G

PLAT004_ALERT_5_G Polymeric Structure Found with Maximum Dimension 2 Info
PLAT432_ALERT_2_G Short Inter X...Y Contact C5 ..C5 . 3.14 Ang.
1-x,y,1/2-z = 2_655 Check

PLAT794_ALERT_5_G Tentative Bond Valency for Zn1 (II) . 2.03 Info
PLAT883_ALERT_1_G Absent Datum for _atom_sites_solution_primary .. Please Do !
PLAT899_ALERT_4_G SHELXL2018 is Outdated and Succeeded by SHELXL 2019/3 Note
PLAT909_ALERT_3_G Percentage of I>2sig(I) Data at Theta(Max) Still 99% Note
PLAT913_ALERT_3_G Missing # of Very Strong Reflections in FCF .... 3 Note
0 2 0, 1 1 2, 2 0 2,
PLAT961_ALERT_5_G Dataset Contains no Negative Intensities .... Please Check
PLAT965_ALERT_2_G The SHELXL WEIGHT Optimisation has not Converged Please Check
PLAT967_ALERT_5_G Note: Two-Theta Cutoff Value in Embedded .res .. 50.5 Degree
PLAT969_ALERT_5_G The 'Henn et al.' R-Factor-gap value .... 3.942 Note
Predicted wR2: Based on SigI**2 1.28 or SHELX Weight 4.63
PLAT978_ALERT_2_G Number C-C Bonds with Positive Residual Density. 4 Info

---

0 **ALERT level A** = Most likely a serious problem - resolve or explain
0 **ALERT level B** = A potentially serious problem, consider carefully
2 **ALERT level C** = Check. Ensure it is not caused by an omission or oversight
12 **ALERT level G** = General information/check it is not something unexpected

1 ALERT type 1 CIF construction/syntax error, inconsistent or missing data
3 ALERT type 2 Indicator that the structure model may be wrong or deficient
4 ALERT type 3 Indicator that the structure quality may be low
1 ALERT type 4 Improvement, methodology, query or suggestion
5 ALERT type 5 Informative message, check

Datablock: 2b

```
Bond precision:       C-C = 0.0085 A              Wavelength=0.71073
Cell:         a=15.7484(11)     b=8.6071(6)       c=16.3698(9)
              alpha=90          beta=117.439(2)   gamma=90
Temperature: 193 K
```

	Calculated	Reported
Volume	1969.3(2)	1969.3(2)
Space group	C c	C c
Hall group	C -2yc	C -2yc
Moiety formula	C18 H22 N4 O8 Zn	C18 H22 N4 O8 Zn
Sum formula	C18 H22 N4 O8 Zn	C18 H22 N4 O8 Zn
Mr	487.79	487.76
Dx,g cm-3	1.645	1.645
Z	4	4
Mu (mm-1)	1.304	1.304
F000	1008.0	1008.0
F000'	1009.60	
h,k,lmax	18,10,19	18,10,19
Nref	3565[1785]	3352
Tmin,Tmax	0.632,0.878	0.256,0.746
Tmin'	0.516	

```
Correction method= # Reported T Limits: Tmin=0.256 Tmax=0.746
AbsCorr = MULTI-SCAN
Data completeness= 1.88/0.94      Theta(max)= 25.249
R(reflections)= 0.0467( 3338)           wR2(reflections)= 0.1014(
                                        3352)
S = 1.250              Npar= 281
```

The following ALERTS were generated. Each ALERT has the format
test-name_ALERT_alert-type_alert-level.
Click on the hyperlinks for more details of the test.

Alert level C

STRVA01_ALERT_4_C Flack test results are ambiguous.
From the CIF: _refine_ls_abs_structure_Flack 0.434
From the CIF: _refine_ls_abs_structure_Flack_su 0.017
PLAT090_ALERT_3_C Poor Data / Parameter Ratio (Zmax > 18) .... 6.31 Note
PLAT094_ALERT_2_C Ratio of Maximum / Minimum Residual Density .... 2.38 Report
PLAT341_ALERT_3_C Low Bond Precision on C-C Bonds .... 0.0085 Ang.
PLAT911_ALERT_3_C Missing FCF Refl Between Thmin & STh/L= 0.600 13 Report
2 0 0, 4 0 0, -5 1 1, 3 1 1, -5 1 2, -3 1 2,
-1 1 3, -2 0 4, -2 0 6, -2 0 8, -10 0 10, -6 0 10,
-2 0 10,
PLAT934_ALERT_3_C Number of (Iobs-Icalc)/Sigma(W) > 10 Outliers .. 1 Check
-1 9 -1,

PLAT971_ALERT_2_C Check Calcd Resid. Dens. 0.87Ang From Zn1 1.73 eA-3
PLAT971_ALERT_2_C Check Calcd Resid. Dens. 0.69Ang From Zn1 1.62 eA-3
PLAT975_ALERT_2_C Check Calcd Resid. Dens. 0.86Ang From O2 . 0.63 eA-3
PLAT975_ALERT_2_C Check Calcd Resid. Dens. 0.59Ang From O5 . 0.56 eA-3

Alert level G

PLAT004_ALERT_5_G Polymeric Structure Found with Maximum Dimension 1 Info
PLAT432_ALERT_2_G Short Inter X...Y Contact O5 ..C11 . 2.92 Ang.
x,1-y,1/2+z = 2_565 Check
PLAT794_ALERT_5_G Tentative Bond Valency for Zn1 (II) . 1.95 Info
PLAT883_ALERT_1_G Absent Datum for _atom_sites_solution_primary .. Please Do !
PLAT899_ALERT_4_G SHELXL2018 is Outdated and Succeeded by SHELXL 2019/3 Note
PLAT909_ALERT_3_G Percentage of I>2sig(I) Data at Theta(Max) Still 99% Note
PLAT913_ALERT_3_G Missing # of Very Strong Reflections in FCF .... 1 Note
-1 1 3,
PLAT961_ALERT_5_G Dataset Contains no Negative Intensities .... Please Check
PLAT965_ALERT_2_G The SHELXL WEIGHT Optimisation has not Converged Please Check
PLAT967_ALERT_5_G Note: Two-Theta Cutoff Value in Embedded .res .. 50.5 Degree
PLAT969_ALERT_5_G The 'Henn et al.' R-Factor-gap value .... 2.681 Note
Predicted wR2: Based on SigI**2 3.78 or SHELX Weight 8.11
PLAT978_ALERT_2_G Number C-C Bonds with Positive Residual Density. 0 Info

0 **ALERT level A** = Most likely a serious problem - resolve or explain
0 **ALERT level B** = A potentially serious problem, consider carefully
10 **ALERT level C** = Check. Ensure it is not caused by an omission or oversight
12 **ALERT level G** = General information/check it is not something unexpected

1 ALERT type 1 CIF construction/syntax error, inconsistent or missing data
8 ALERT type 2 Indicator that the structure model may be wrong or deficient
6 ALERT type 3 Indicator that the structure quality may be low
2 ALERT type 4 Improvement, methodology, query or suggestion
5 ALERT type 5 Informative message, check

Datablock: 2c

Bond precision:	C-C = 0.0055 A		Wavelength=0.71073
Cell:	a=9.1488(2)	b=9.1488(2)	c=35.5558(15)
	alpha=90	beta=90	gamma=120
Temperature: 193 K			

	Calculated	Reported
Volume	2577.33(16)	2577.33(16)
Space group	P 32 2 1	P 32 2 1
Hall group	P 32 2"	P 32 2"
Moiety formula	C30 H50 N4 O10 Zn	C30 H50 N4 O10 Zn
Sum formula	C30 H50 N4 O10 Zn	C30 H50 N4 O10 Zn
Mr	692.13	692.11
Dx,g cm-3	1.338	1.338
Z	3	3
Mu (mm-1)	0.773	0.773
F000	1104.0	1104.0
F000'	1105.34	
h,k,lmax	10,10,42	10,10,42
Nref	3081[1846]	3072
Tmin,Tmax	0.831,0.926	0.646,0.746
Tmin'	0.763	

Correction method= # Reported T Limits: Tmin=0.646 Tmax=0.746
AbsCorr = MULTI-SCAN

Data completeness= 1.66/1.00 Theta(max)= 25.235

R(reflections)= 0.0287(3042) wR2(reflections)= 0.0771(3072)

S = 1.116 Npar= 209

The following ALERTS were generated. Each ALERT has the format
test-name_ALERT_alert-type_alert-level.
Click on the hyperlinks for more details of the test.

Alert level C

PLAT220_ALERT_2_C NonSolvent Resd 1 C Ueq(max)/Ueq(min) Range 4.2 Ratio
PLAT222_ALERT_3_C NonSolvent Resd 1 H Uiso(max)/Uiso(min) Range 4.5 Ratio
PLAT242_ALERT_2_C Low 'MainMol' Ueq as Compared to Neighbors of C7 Check

PLAT415_ALERT_2_C Short Inter D-H..H-X H1O5 ..H3 . 2.13 Ang.
1+x,y,z = 1_655 Check
PLAT415_ALERT_2_C Short Inter D-H..H-X H1O5 ..H9B . 2.06 Ang.
1+y,-1+x,1-z = 4_646 Check
PLAT415_ALERT_2_C Short Inter D-H..H-X H2 ..H2O5 . 2.07 Ang.
-1+x-y,-y,4/3-z = 5_456 Check
PLAT934_ALERT_3_C Number of (Iobs-Icalc)/Sigma(W) > 10 Outliers .. 1 Check
-4 4 1,
PLAT976_ALERT_2_C Check Calcd Resid. Dens. 1.03Ang From O5 . -0.41 eA-3

Alert level G

PLAT003_ALERT_2_G Number of Uiso or U(i,j) Restrained non-H-Atoms 4 Report
PLAT004_ALERT_5_G Polymeric Structure Found with Maximum Dimension 2 Info
PLAT007_ALERT_5_G Number of Unrefined Donor-H Atoms .... 2 Report
H1O5 H2O5
PLAT033_ALERT_4_G Flack x Value Deviates > 3.0 * sigma from Zero . 0.079 Note
PLAT177_ALERT_4_G The CIF-Embedded .res File Contains DELU Records 1 Report
PLAT178_ALERT_4_G The CIF-Embedded .res File Contains SIMU Records 1 Report
PLAT186_ALERT_4_G The CIF-Embedded .res File Contains ISOR Records 1 Report
PLAT188_ALERT_3_G A Non-default SIMU Restraint Value has been used 0.0050 Report
PLAT192_ALERT_3_G A Non-default DELU Restraint Value for First Par 0.0050 Report
PLAT720_ALERT_4_G Number of Unusual/Non-Standard Labels .... 2 Note
H1O5 H2O5
PLAT794_ALERT_5_G Tentative Bond Valency for Zn1 (II) . 1.99 Info
PLAT860_ALERT_3_G Number of Least-Squares Restraints .... 24 Note
PLAT883_ALERT_1_G Absent Datum for _atom_sites_solution_primary .. Please Do !
PLAT899_ALERT_4_G SHELXL2018 is Outdated and Succeeded by SHELXL 2019/3 Note
PLAT909_ALERT_3_G Percentage of I>2sig(I) Data at Theta(Max) Still 99% Note
PLAT910_ALERT_3_G Missing # of FCF Reflection(s) Below Theta(Min). 1 Note
0 0 3,
PLAT913_ALERT_3_G Missing # of Very Strong Reflections in FCF .... 1 Note
0 0 3,
PLAT961_ALERT_5_G Dataset Contains no Negative Intensities .... Please Check
PLAT967_ALERT_5_G Note: Two-Theta Cutoff Value in Embedded .res .. 50.5 Degree
PLAT969_ALERT_5_G The 'Henn et al.' R-Factor-gap value .... 3.610 Note
Predicted wR2: Based on SigI**2 2.14 or SHELX Weight 6.91
PLAT978_ALERT_2_G Number C-C Bonds with Positive Residual Density. 2 Info

0 **ALERT level A** = Most likely a serious problem - resolve or explain
0 **ALERT level B** = A potentially serious problem, consider carefully
8 **ALERT level C** = Check. Ensure it is not caused by an omission or oversight
21 **ALERT level G** = General information/check it is not something unexpected

1 ALERT type 1 CIF construction/syntax error, inconsistent or missing data
8 ALERT type 2 Indicator that the structure model may be wrong or deficient
8 ALERT type 3 Indicator that the structure quality may be low
6 ALERT type 4 Improvement, methodology, query or suggestion
6 ALERT type 5 Informative message, check

Datablock: 2d

Bond precision:	C-C = 0.0058 A		Wavelength=0.71073
Cell:	a=10.6171(11)	b=9.6392(10)	c=11.0506(12)
	alpha=90	beta=108.895(4)	gamma=90
Temperature:	193 K		

	Calculated	Reported
Volume	1070.0(2)	1070.0(2)
Space group	P 21	P 1 21 1
Hall group	P 2yb	P 2yb
Moiety formula	C18 H31 N3 O7 Zn	C18 H31 N3 O7 Zn
Sum formula	C18 H31 N3 O7 Zn	C18 H31 N3 O7 Zn
Mr	466.85	466.83
Dx,g cm-3	1.449	1.449
Z	2	2
Mu (mm-1)	1.191	1.191
F000	492.0	492.0
F000'	492.78	
h,k,lmax	12,11,13	12,11,13
Nref	3875[2064]	3699
Tmin,Tmax	0.842,0.888	0.581,0.746
Tmin'	0.551	

```
Correction method= # Reported T Limits: Tmin=0.581 Tmax=0.746
AbsCorr = MULTI-SCAN
Data completeness= 1.79/0.95       Theta(max)= 25.245
R(reflections)= 0.0286( 3570)            wR2(reflections)= 0.0748( 3699)
S = 1.061             Npar= 270
```

The following ALERTS were generated. Each ALERT has the format
test-name_ALERT_alert-type_alert-level.
Click on the hyperlinks for more details of the test.

Alert level C

PLAT029_ALERT_3_C _diffrn_measured_fraction_theta_full value Low . 0.970 Why?
PLAT090_ALERT_3_C Poor Data / Parameter Ratio (Zmax > 18) .... 7.42 Note
PLAT911_ALERT_3_C Missing FCF Refl Between Thmin & STh/L= 0.600 57 Report
0 2 0, 2 0 0, 2 1 0, 3 1 0, 4 0 0, 12 0 0,
-12 0 1, -4 0 1, -3 0 1, -2 0 1, -2 1 1, -1 1 1,
1 0 1, 2 0 1, 2 1 1, 3 1 1, 4 0 1, 5 0 1,
6 0 1, -5 0 2, -4 1 2, 2 1 2, 2 3 2, 3 1 2,
4 3 2, 5 2 2, -5 2 3, -3 9 3, 6 0 3, 7 0 3,
7 1 3, 8 1 3, 2 0 4, 5 0 4, 6 0 4, 7 0 4,
7 1 4, 8 0 4, 8 1 4, 9 0 4, 9 2 4, 0 2 5,
2 0 5, 6 0 5, 9 1 5, -1 9 6, 6 4 6, 8 2 6,
7 0 7, 7 2 7, 1 0 8, 3 0 8, 6 0 8, 5 0 9,
-3 4 12, -3 2 13, -2 0 13,
PLAT913_ALERT_3_C Missing # of Very Strong Reflections in FCF .... 4 Note
0 2 0, 3 1 0, -2 1 1, 0 0 1,

Alert level G

PLAT004_ALERT_5_G Polymeric Structure Found with Maximum Dimension 1 Info
PLAT007_ALERT_5_G Number of Unrefined Donor-H Atoms .... 4 Report
H3A H3B H7A H7B
PLAT033_ALERT_4_G Flack x Value Deviates > 3.0 * sigma from Zero . 0.109 Note
PLAT794_ALERT_5_G Tentative Bond Valency for Zn1 (II) . 1.99 Info
PLAT909_ALERT_3_G Percentage of I>2sig(I) Data at Theta(Max) Still 92% Note
PLAT910_ALERT_3_G Missing # of FCF Reflection(s) Below Theta(Min). 4 Note
1 0 0, -1 0 1, 0 0 1, 0 1 1,
PLAT933_ALERT_2_G Number of HKL-OMIT Records in Embedded .res File 20 Note
-5 -2 3, -5 0 2, -4 0 1, -3 0 1, -3 9 3, -2 0 1,
0 -2 0, 1 0 0, 2 0 0, 2 0 1, 2 0 4, 2 0 5,
3 -1 0, 3 -1 1, 3 1 0, 3 1 1, 3 2 0, 4 0 0,
4 0 1, 5 2 2,
PLAT961_ALERT_5_G Dataset Contains no Negative Intensities .... Please Check
PLAT967_ALERT_5_G Note: Two-Theta Cutoff Value in Embedded .res .. 50.5 Degree
PLAT969_ALERT_5_G The 'Henn et al.' R-Factor-gap value .... 1.583 Note
Predicted wR2: Based on SigI**2 4.72 or SHELX Weight 7.05
PLAT978_ALERT_2_G Number C-C Bonds with Positive Residual Density. 2 Info

---

0 **ALERT level A** = Most likely a serious problem - resolve or explain
0 **ALERT level B** = A potentially serious problem, consider carefully
4 **ALERT level C** = Check. Ensure it is not caused by an omission or oversight
11 **ALERT level G** = General information/check it is not something unexpected

0 ALERT type 1 CIF construction/syntax error, inconsistent or missing data
2 ALERT type 2 Indicator that the structure model may be wrong or deficient
6 ALERT type 3 Indicator that the structure quality may be low
1 ALERT type 4 Improvement, methodology, query or suggestion
6 ALERT type 5 Informative message, check

Datablock: 2e

```
Bond precision:      C-C = 0.0192 A              Wavelength=0.71073
Cell:       a=27.801(4)      b=9.8432(10)     c=10.9492(14)
            alpha=90         beta=108.040(6)  gamma=90
Temperature: 193 K
```

	Calculated	Reported
Volume	2849.0(6)	2848.9(6)
Space group	C 2	C 1 2 1
Hall group	C 2y	C 2y
Moiety formula	C21 H35.17 N3 O7 Zn [+ solvent]	C21 H35.169 N3 O7 Zn, 0.5[]
Sum formula	C21 H35.17 N3 O7 Zn [+ solvent]	C21 H35.17 N3 O7 Zn

Mr	507.08	507.06
Dx,g cm-3	1.182	1.182
Z	4	4
Mu (mm-1)	0.900	0.900
F000	1072.7	1073.0
F000'	1074.27	
h,k,lmax	33,11,13	33,11,13
Nref	5147[2738]	5102
Tmin,Tmax	0.730,0.914	0.661,0.746
Tmin'	0.631	

Correction method= # Reported T Limits: Tmin=0.661 Tmax=0.746
AbsCorr = MULTI-SCAN

Data completeness= 1.86/0.99 Theta(max)= 25.248

R(reflections)= 0.0842(4926) wR2(reflections)= 0.2418(5102)

S = 1.147 Npar= 344

The following ALERTS were generated. Each ALERT has the format
test-name_ALERT_alert-type_alert-level.
Click on the hyperlinks for more details of the test.

Alert level B

PLAT341_ALERT_3_B Low Bond Precision on C-C Bonds .... 0.01918 Ang.
PLAT420_ALERT_2_B D-H Bond Without Acceptor O7 --H7B . Please Check

---

Alert level C

PLAT090_ALERT_3_C Poor Data / Parameter Ratio (Zmax > 18) .... 7.94 Note
PLAT911_ALERT_3_C Missing FCF Refl Between Thmin & STh/L= 0.600 4 Report
0 2 0, 3 3 0, -1 3 2, 1 3 3,
PLAT918_ALERT_3_C Reflection(s) with I(obs) much Smaller I(calc) . 14 Check
PLAT971_ALERT_2_C Check Calcd Resid. Dens. 3.13Ang From C9 1.74 eA-3
PLAT971_ALERT_2_C Check Calcd Resid. Dens. 0.59Ang From C14A 1.72 eA-3
PLAT977_ALERT_2_C Check Negative Difference Density on H9A . -0.42 eA-3

Alert level G

PLAT002_ALERT_2_G Number of Distance or Angle Restraints on AtSite 12 Note
PLAT003_ALERT_2_G Number of Uiso or U(i,j) Restrained non-H-Atoms 12 Report
PLAT004_ALERT_5_G Polymeric Structure Found with Maximum Dimension 1 Info
PLAT007_ALERT_5_G Number of Unrefined Donor-H Atoms .... 4 Report
H3A H3B H7A H7B
PLAT042_ALERT_1_G Calc. and Reported MoietyFormula Strings Differ Please Check
Calc: C21 H35.17 N3 O7 Zn
Rep.: C21 H35.169 N3 O7 Zn, 0.5[]
PLAT072_ALERT_2_G SHELXL First Parameter in WGHT Unusually Large 0.18 Report
PLAT083_ALERT_2_G SHELXL Second Parameter in WGHT Unusually Large 8.12 Why ?
PLAT128_ALERT_4_G Alternate Setting for Input Space Group C2 I2 Note
PLAT172_ALERT_4_G The CIF-Embedded .res File Contains DFIX Records 4 Report
PLAT177_ALERT_4_G The CIF-Embedded .res File Contains DELU Records 1 Report
PLAT178_ALERT_4_G The CIF-Embedded .res File Contains SIMU Records 1 Report
PLAT186_ALERT_4_G The CIF-Embedded .res File Contains ISOR Records 1 Report
PLAT188_ALERT_3_G A Non-default SIMU Restraint Value has been used 0.0050 Report
PLAT192_ALERT_3_G A Non-default DELU Restraint Value for First Par 0.0050 Report
PLAT192_ALERT_3_G A Non-default DELU Restraint Value for SecondPar 0.0050 Report
PLAT301_ALERT_3_G Main Residue Disorder ....(Resd 1) 16% Note
PLAT315_ALERT_2_G Singly Bonded Carbon Detected (H-atoms Missing). C15B Check
PLAT412_ALERT_2_G Short Intra XH3 .. XHn H7D ..H15C . 2.07 Ang.
3/2-x,1/2+y,1-z = 4_656 Check
PLAT412_ALERT_2_G Short Intra XH3 .. XHn H21A ..H14A . 1.64 Ang.
3/2-x,1/2+y,1-z = 4_656 Check
PLAT412_ALERT_2_G Short Intra XH3 .. XHn H21A ..H14B . 2.12 Ang.
3/2-x,1/2+y,1-z = 4_656 Check
PLAT414_ALERT_2_G Short Intra D-H..H-X H3B ..H14A . 1.95 Ang.
3/2-x,1/2+y,1-z = 4_656 Check
PLAT432_ALERT_2_G Short Inter X...Y Contact O5 ..C3 . 3.02 Ang.
x,y,1+z = 1_556 Check
PLAT605_ALERT_4_G Largest Solvent Accessible VOID in the Structure 247 A**3
PLAT860_ALERT_3_G Number of Least-Squares Restraints .... 221 Note
PLAT869_ALERT_4_G ALERTS Related to the Use of SQUEEZE Suppressed ! Info
PLAT909_ALERT_3_G Percentage of I>2sig(I) Data at Theta(Max) Still 91% Note
PLAT910_ALERT_3_G Missing # of FCF Reflection(s) Below Theta(Min). 1 Note
2 0 0,
PLAT913_ALERT_3_G Missing # of Very Strong Reflections in FCF .... 1 Note

2 0 0,
PLAT933_ALERT_2_G Number of HKL-OMIT Records in Embedded .res File 6 Note
-1 3 2, 1 -3 3, 1 3 2, 1 3 3, 3 -3 0, 3 3 0,
PLAT961_ALERT_5_G Dataset Contains no Negative Intensities .... Please Check
PLAT967_ALERT_5_G Note: Two-Theta Cutoff Value in Embedded .res .. 50.5 Degree
PLAT969_ALERT_5_G The 'Henn et al.' R-Factor-gap value .... 8.955 Note
Predicted wR2: Based on SigI**2 2.70 or SHELX Weight 21.17
PLAT978_ALERT_2_G Number C-C Bonds with Positive Residual Density. 1 Info

---

0 **ALERT level A** = Most likely a serious problem - resolve or explain
2 **ALERT level B** = A potentially serious problem, consider carefully
6 **ALERT level C** = Check. Ensure it is not caused by an omission or oversight
33 **ALERT level G** = General information/check it is not something unexpected

1 ALERT type 1 CIF construction/syntax error, inconsistent or missing data
16 ALERT type 2 Indicator that the structure model may be wrong or deficient
12 ALERT type 3 Indicator that the structure quality may be low
7 ALERT type 4 Improvement, methodology, query or suggestion
5 ALERT type 5 Informative message, check

It is advisable to attempt to resolve as many as possible of the alerts in all categories. Often the minor alerts point to easily fixed oversights, errors and omissions in your CIF or refinement strategy, so attention to these fine details can be worthwhile. In order to resolve some of the more serious problems it may be necessary to carry out additional measurements or structure refinements. However, the purpose of your study may justify the reported deviations and the more serious of these should normally be commented upon in the discussion or experimental section of a paper or in the "special_details" fields of the CIF. checkCIF was carefully designed to identify outliers and unusual parameters, but every test has its limitations and alerts that are not important in a particular case may appear. Conversely, the absence of alerts does not guarantee there are no aspects of the results needing attention. It is up to the individual to critically assess their own results and, if necessary, seek expert advice.

Publication of your CIF in IUCr journals

A basic structural check has been run on your CIF. These basic checks will be run on all CIFs submitted for publication in IUCr journals (*Acta Crystallographica*, *Journal of Applied Crystallography*, *Journal of Synchrotron Radiation*); however, if you intend to submit to *Acta Crystallographica Section C* or *E* or *IUCrData*, you should make sure that full publication checks are run on the final version of your CIF prior to submission.

Publication of your CIF in other journals

Please refer to the *Notes for Authors* of the relevant journal for any special instructions relating to CIF submission.

PLATON version of 11/11/2024; check.def file version of 11/11/2024

Datablock 2a - ellipsoid plot

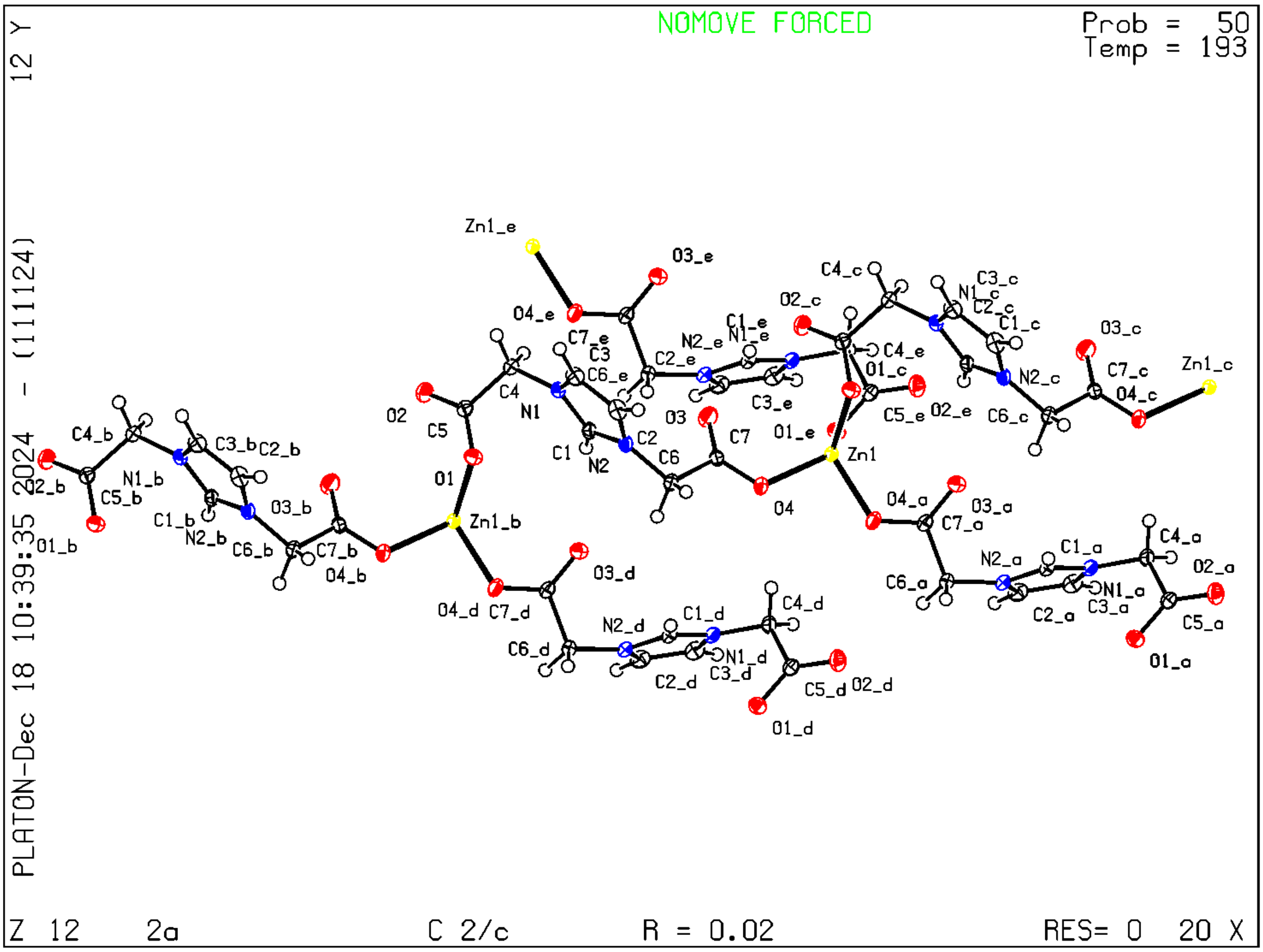

Datablock 2b - ellipsoid plot

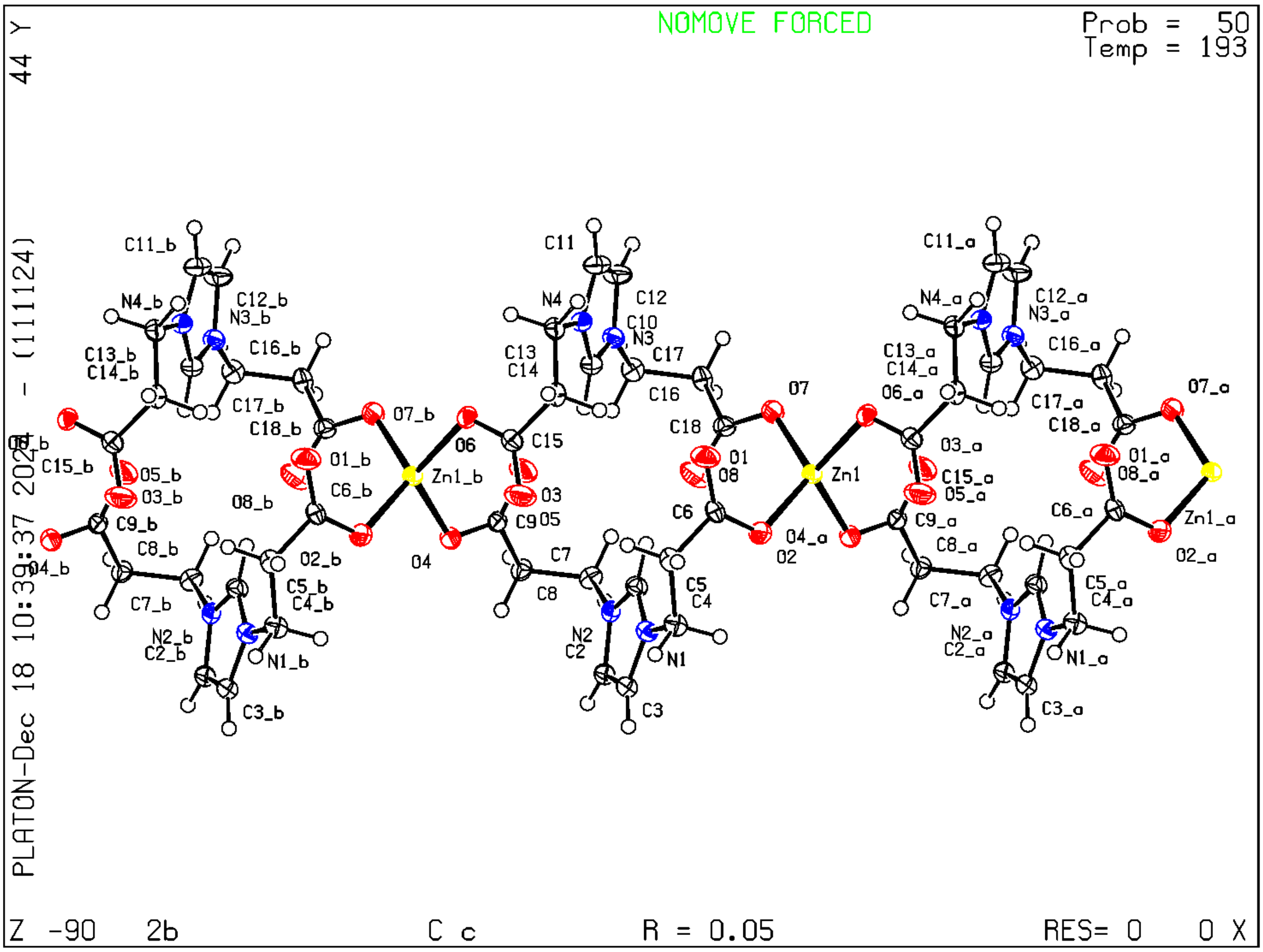

Datablock 2c - ellipsoid plot

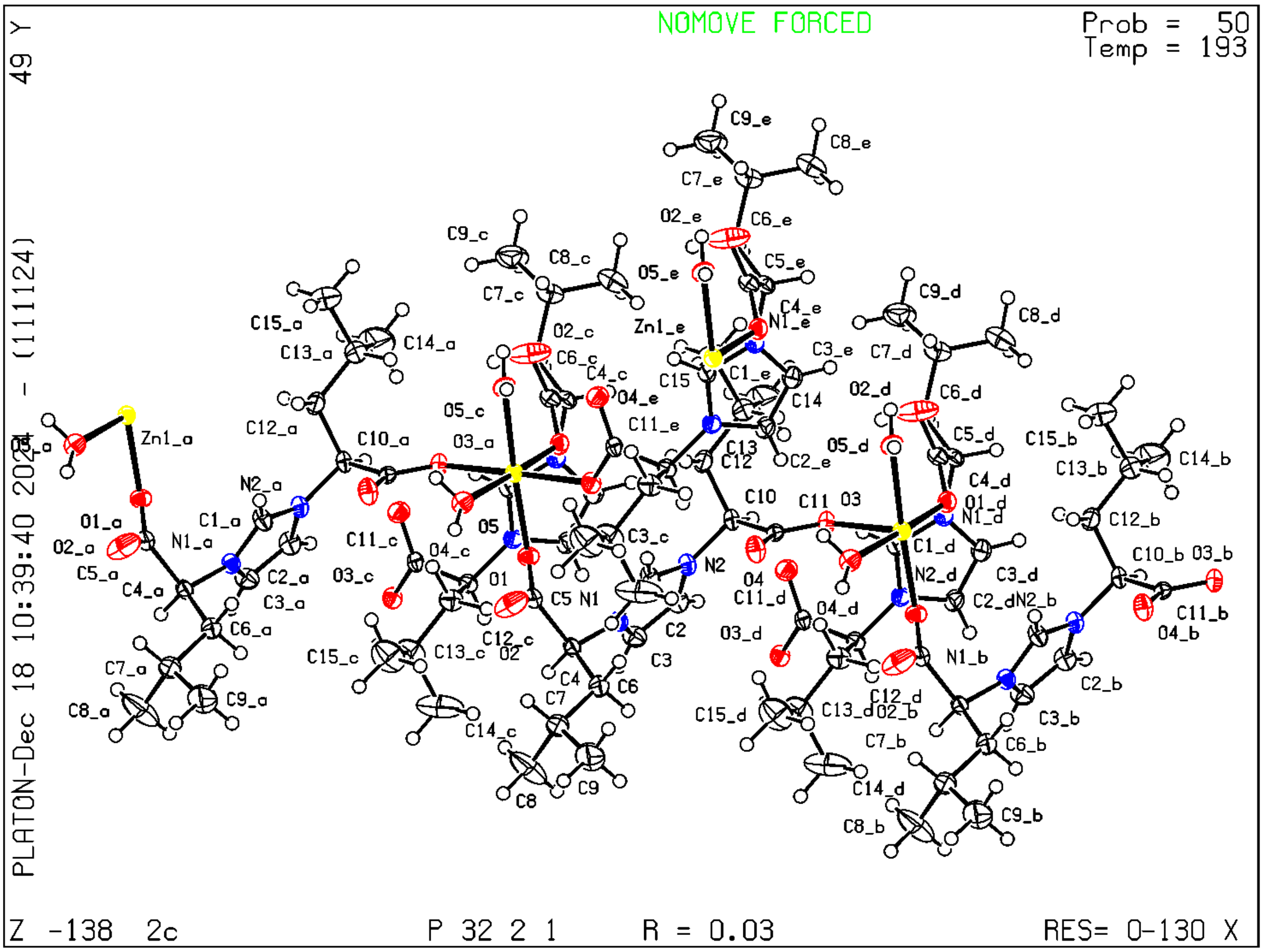

Datablock 2d - ellipsoid plot

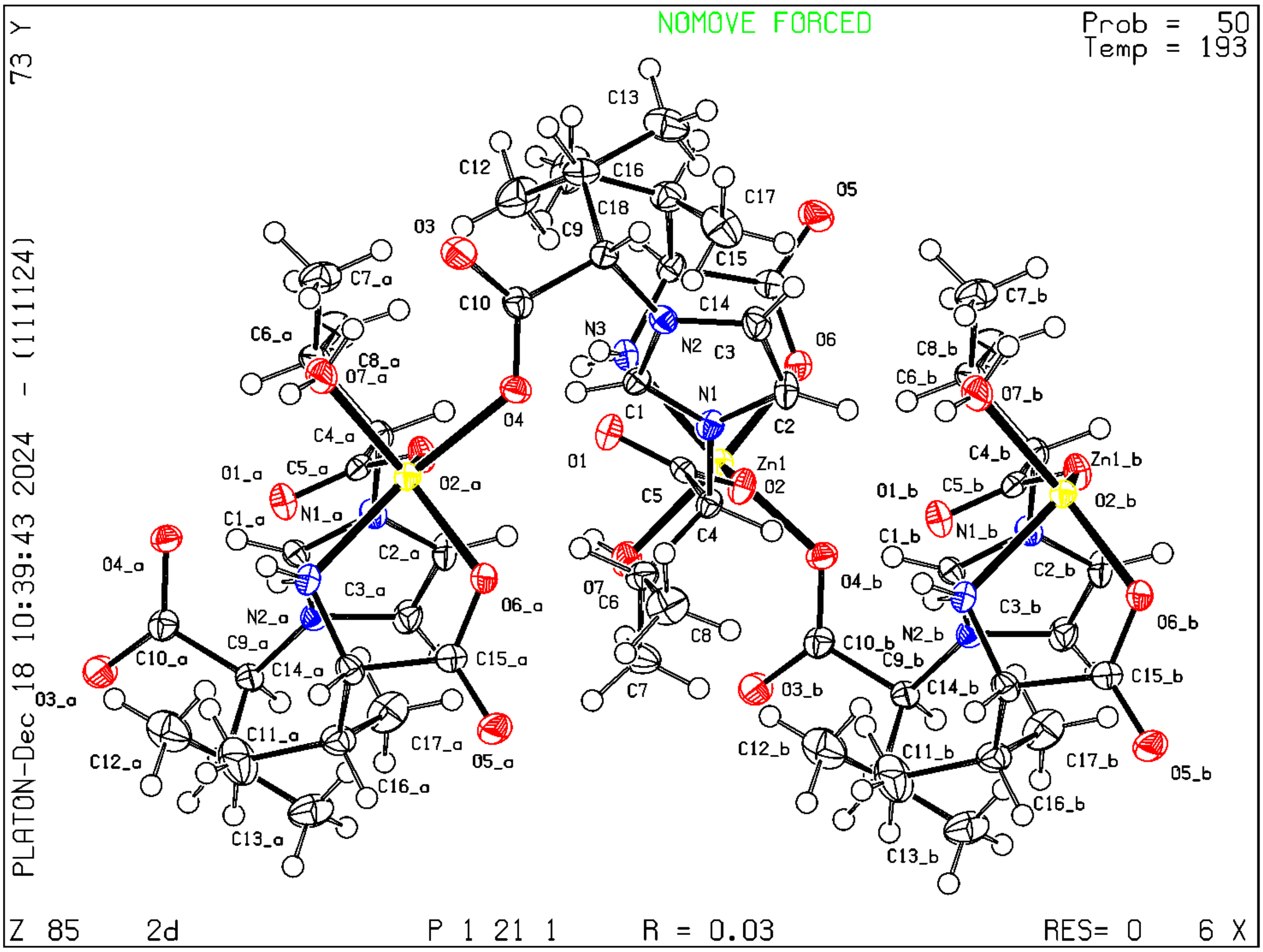

Datablock 2e - ellipsoid plot

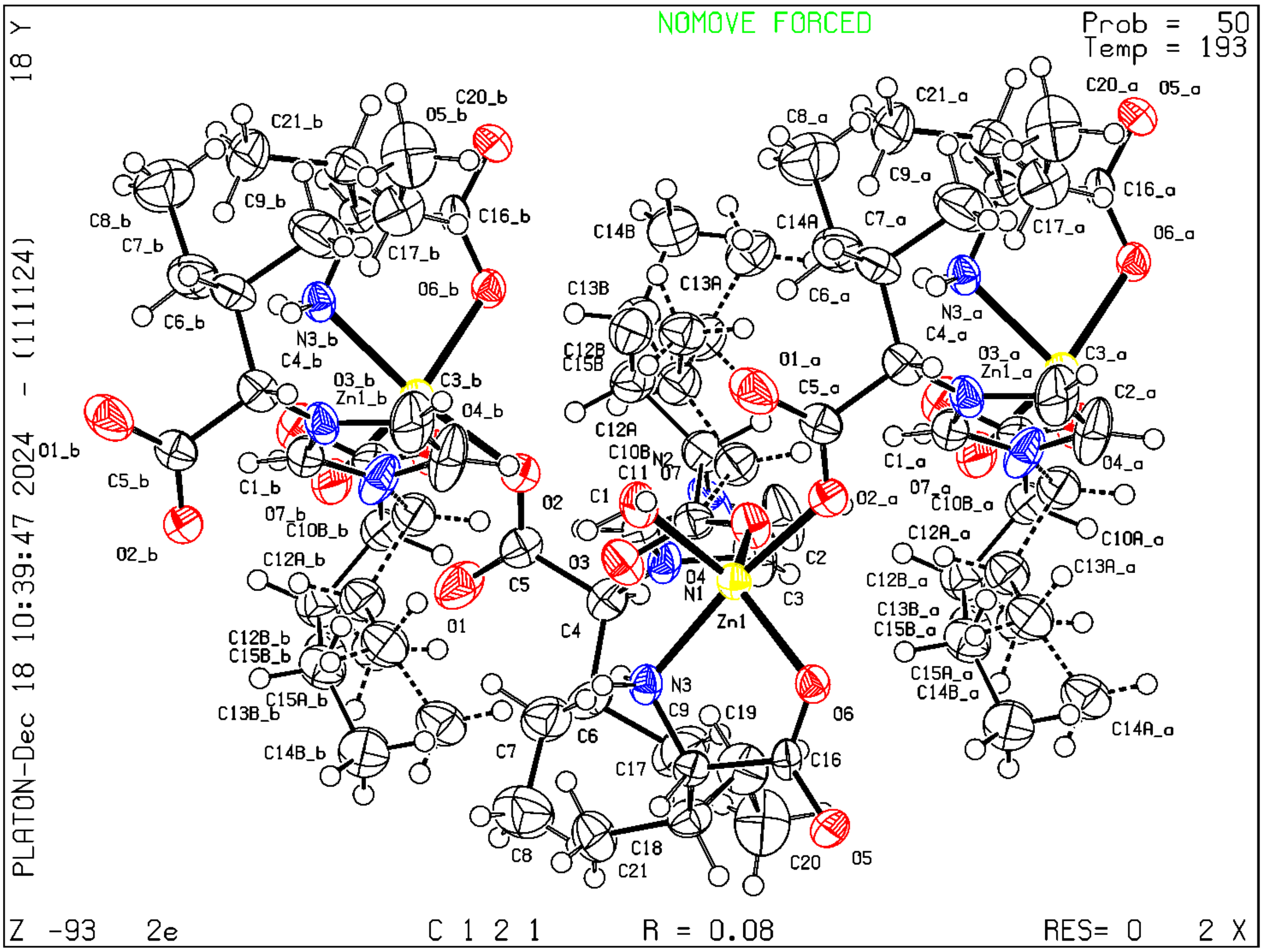

Download CIF editor (publCIF) from the IUCr
Download CIF editor (enCIFer) from the CCDC
Test a new CIF entry
