# Supplementary material for: Synthesis, Characterization, and Preliminary In Vitro Anticancer Activity of Zinc Complexes Containing Amino Acid-Derived Imidazolium-Based Dicarboxylate Ligands
Source: Int J Mol Sci. 2025 Mar 30;26(7):3202. doi: 10.3390/ijms26073202 (PMC11989707; doi:10.3390/ijms26073202)

SUPPLEMENTARY MATERIAL

Synthesis, Characterization and Selective Anticancer Activity of Zinc Complexes containing Amino Acid-derived Imidazolium-based Dicarboxylate Ligands

Carlos J. Carrasco, [1] Antonio Pastor, [1] María del Mar Conejo, [1] Eleuterio Álvarez, [2] José Manuel Calderón-Montaño, [3] Miguel López-Lázaro, [3] and Agustín Galindo* [1]

[1] Departamento de Química Inorgánica, Facultad de Química, Universidad de Sevilla, 41071 Sevilla, Spain.
[2] Instituto de Investigaciones Químicas, CSIC-Universidad de Sevilla, Avda. Américo Vespucio 49, 41092 Sevilla, Spain.
[3] Departamento de Farmacología, Facultad de Farmacia, Universidad de Sevilla, Sevilla, Spain.

Spectroscopical characterization of complexes 2a-2e……………….…………S3-S10

Figure S1. FTIR and NMR (^{1}H and ^{13}C{^{1}H}) spectra of complexes **2a**-**2e**.

X-ray structural characterization of complexes 2a-2e…………………………....S11-S21

Figure S2. Non-classical C=O···H-C hydrogen bonds observed between adjacent 1D chains in **2b**.

Figure S3. 3D crystal packing of complex **2b** viewed along *b* axis.

Figure S4. Complex **2c**: (a) 2D metal–organic framework; (b) 3D packing viewed along *a* axis; (c) square lattice topology.

Figure S5. Complex **2d**: (a) 1D coordination polymer along *b* axis viewed along *a* axis. (b) 3D packing viewed along *b* axis.

Table S1. Crystal data and structure refinement for complexes **2a**-**2e**.

Table S2. Selected structural parameters (bond lengths, Å; angles, °) for zinc complexes **2**.

Figure S6. Complex **2a**: (a) asymmetric unit of complex, (b) polymeric growing, (c) 3D packing viewed along *b* axis, (d) 3D packing viewed along *a* axis.

Anticancer activity……………………………………………………………………S22

Table S3. IC_{50} values (μM) and selectivity indexes (SI) of common anticancer drugs tested against human cell lines.

Figure S7. Effect of HL^R compounds **1** (R = Gly, βAla, Val, Leu, and Ile) on the viability of human non-malignant cells (HaCaT) and human cancer cells (A549, MeWo and T24).

Figure S8. Representative photographs of untreated cells (control), cells treated with positive controls carboplatin and gemcitabine, and cells exposed to complexes **2a** and **2c**.

Figure S1. FTIR and NMR (^{1}H and ^{13}C{^{1}H}) spectra of complexes **2a**-**2e**.

FTIR spectra

2a

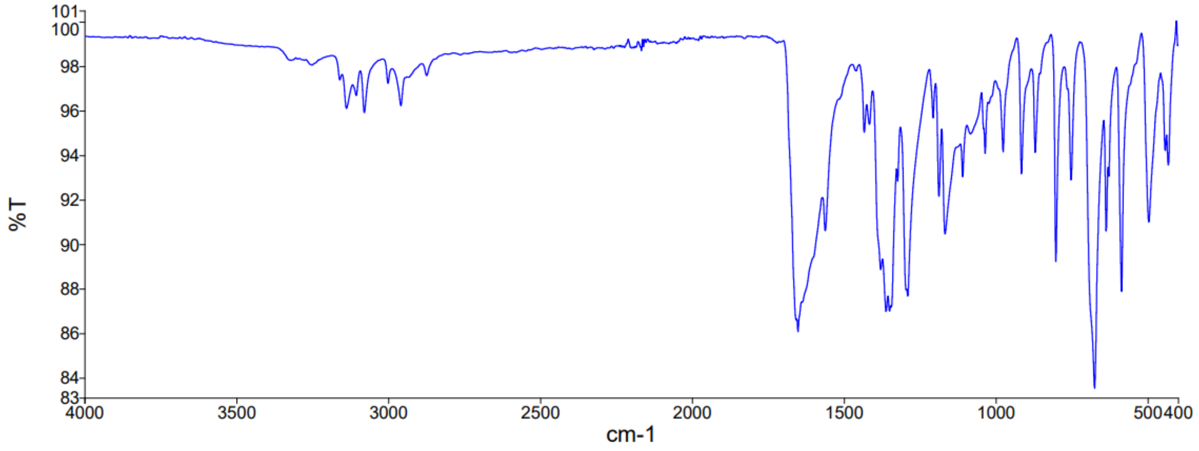

2b

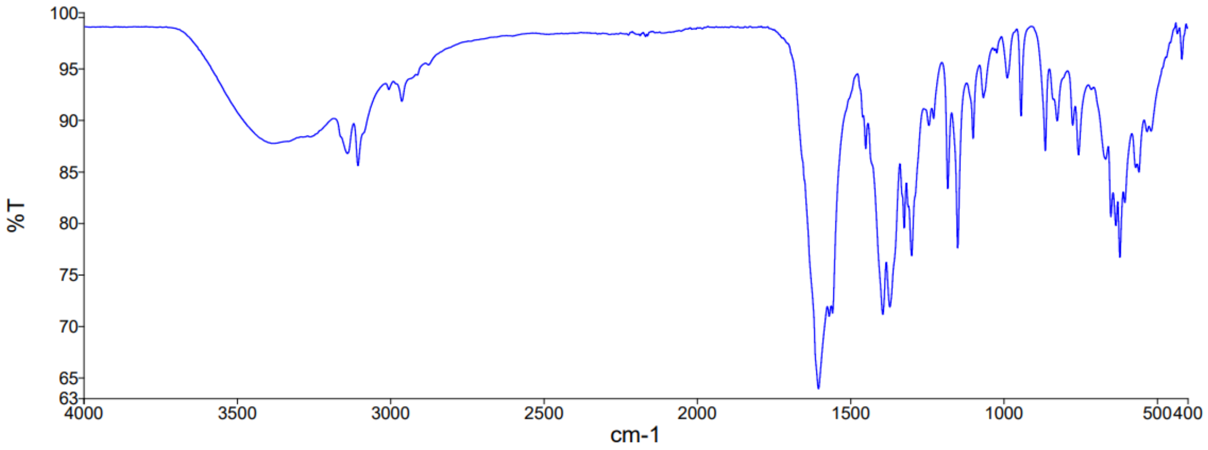

2c

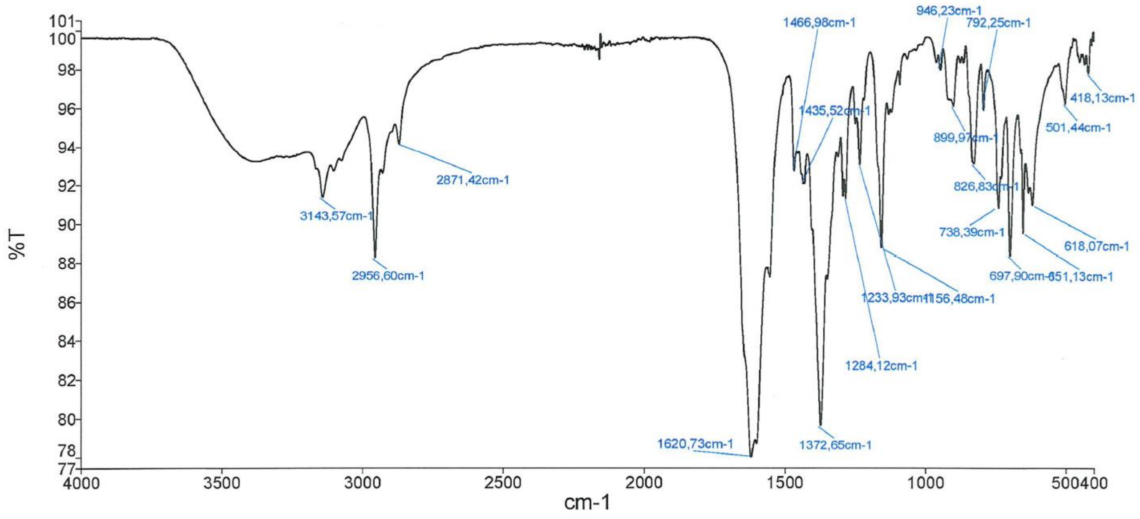

%T
cm-1
3143,57cm-1
2956,60cm-1
2871,42cm-1
1620,73cm-1
1466,98cm-1
1435,52cm-1
1372,65cm-1
1284,12cm-1
1233,93cm-1
1156,48cm-1
946,23cm-1
899,97cm-1
826,83cm-1
792,25cm-1
738,39cm-1
697,90cm-1
651,13cm-1
618,07cm-1
501,44cm-1
418,13cm-1

2d

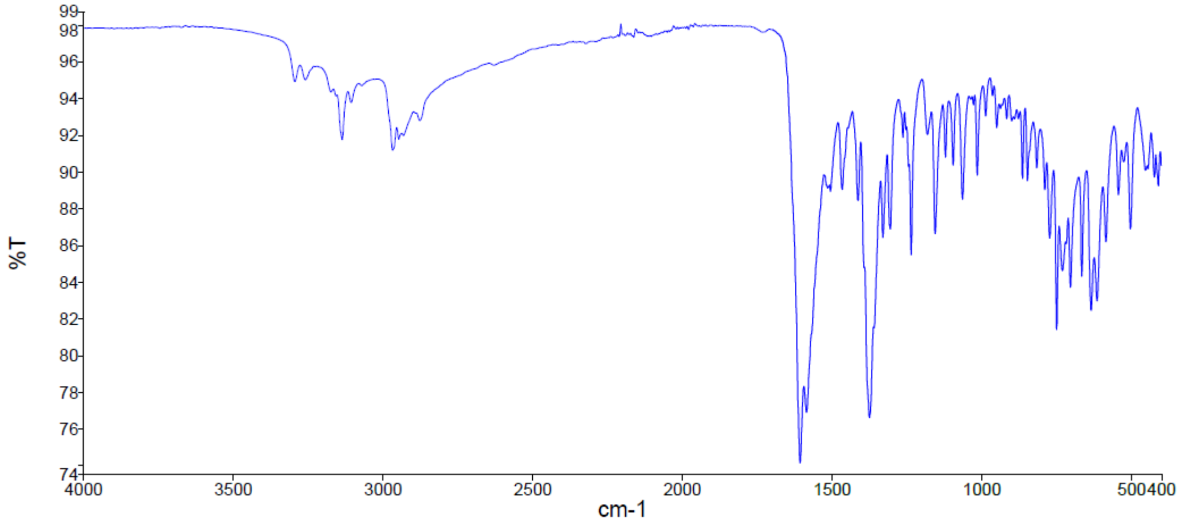

%T
cm-1

2e

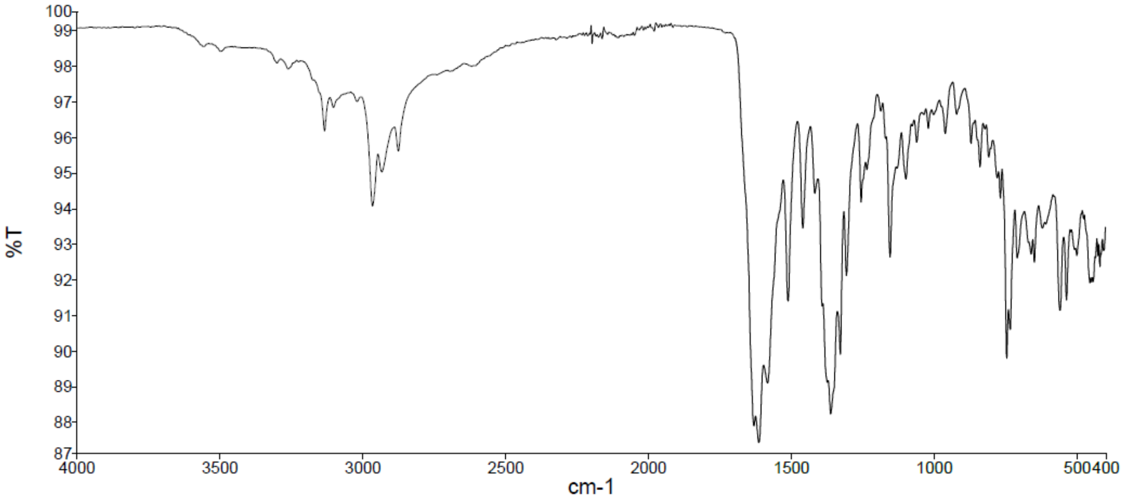

^{1}H NMR spectra

2a

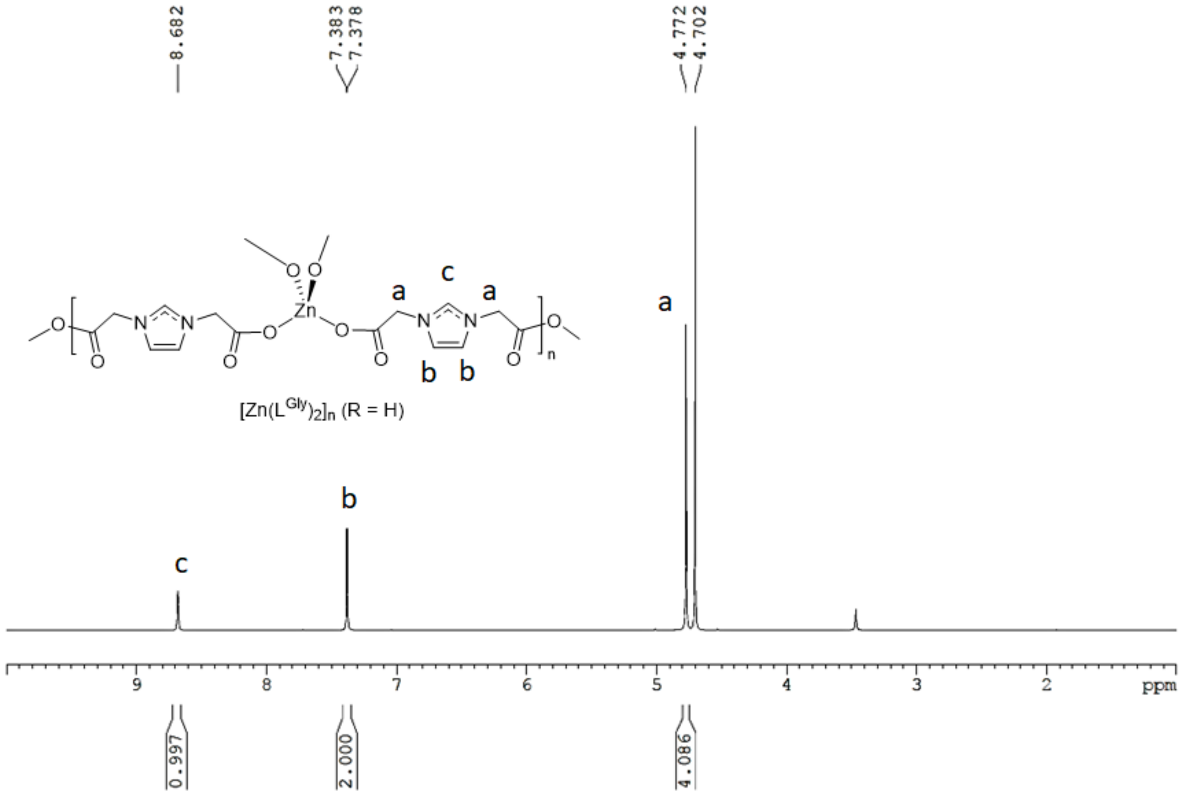

2b

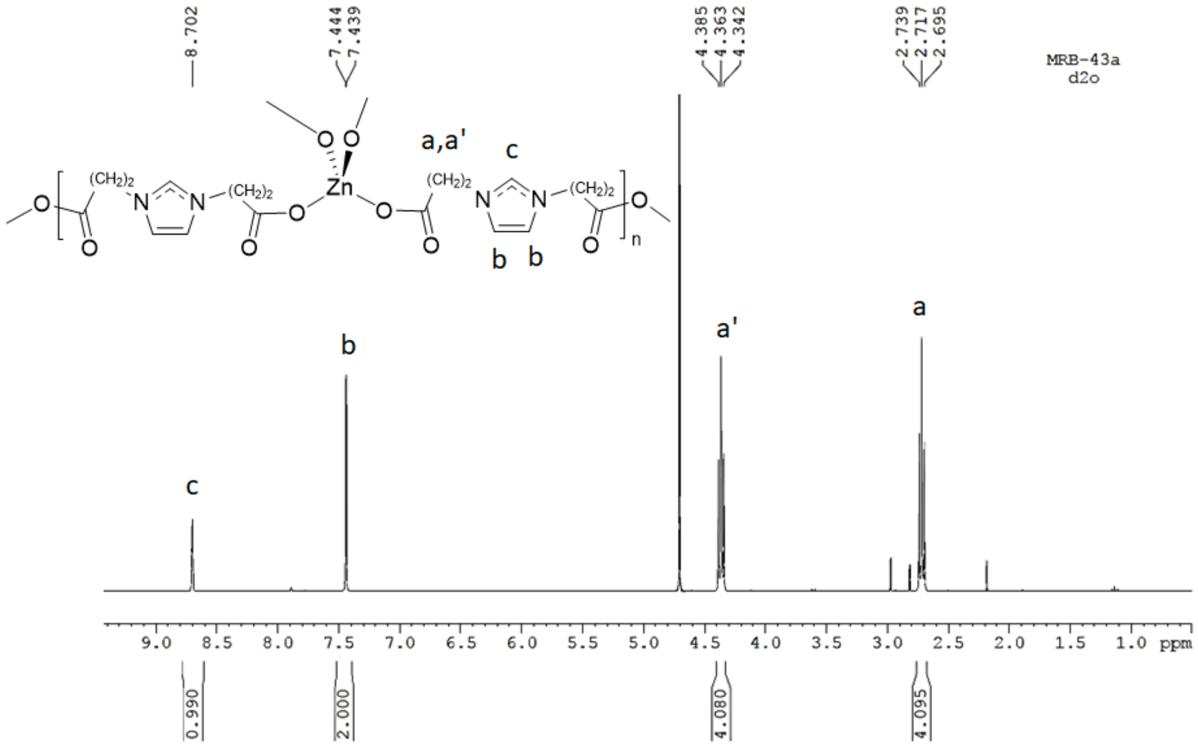
8.702
7.444
7.439
4.385
4.363
4.342
2.739
2.717
2.695
MRB-43a
d2o
a,a'
c
b b
n
c
b
a'
a
ppm
0.990
2.000
4.080
4.095

2c

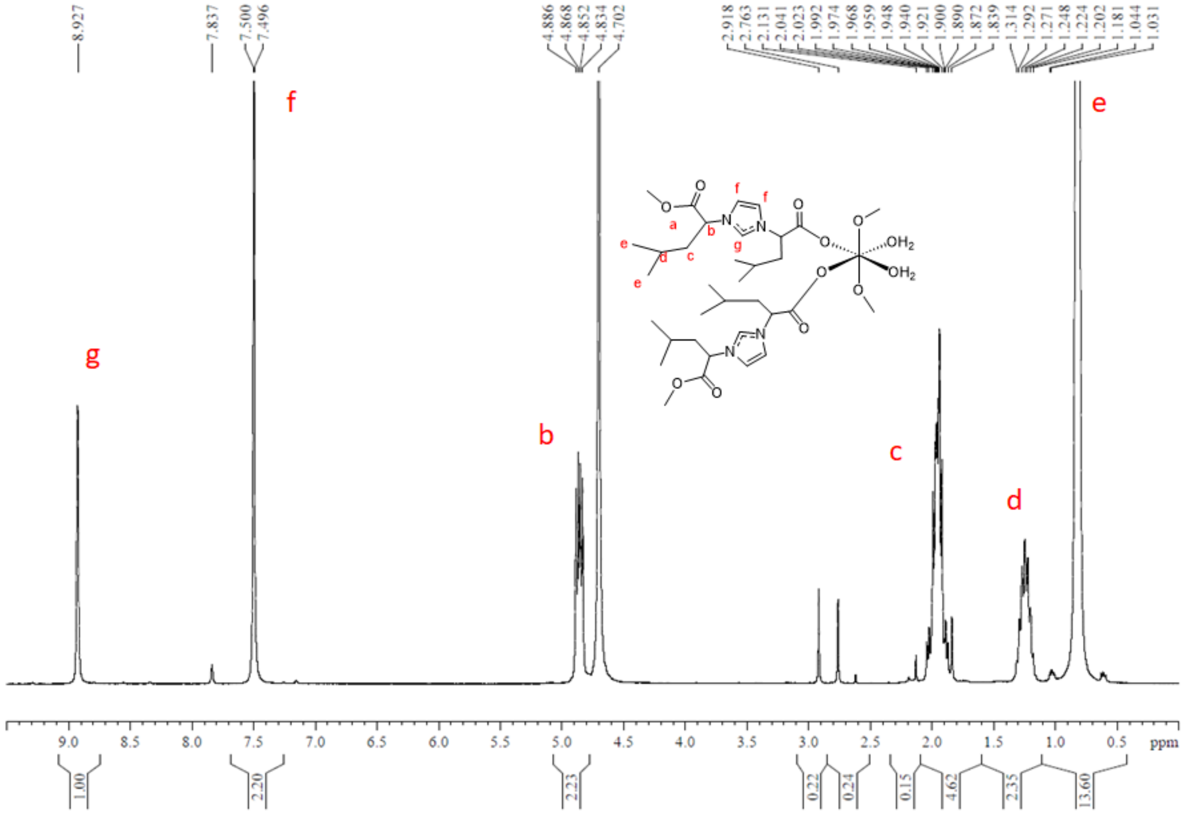
8.927
7.837
7.500
7.496
4.886
4.868
4.852
4.834
4.702
2.918
2.763
2.131
2.041
2.023
1.992
1.974
1.968
1.959
1.948
1.940
1.921
1.900
1.890
1.872
1.839
1.314
1.292
1.271
1.248
1.224
1.202
1.181
1.044
1.031
f
e
g
b
c
d
ppm
1.00
2.20
2.23
0.22
0.24
0.15
4.62
2.35
13.60

2d

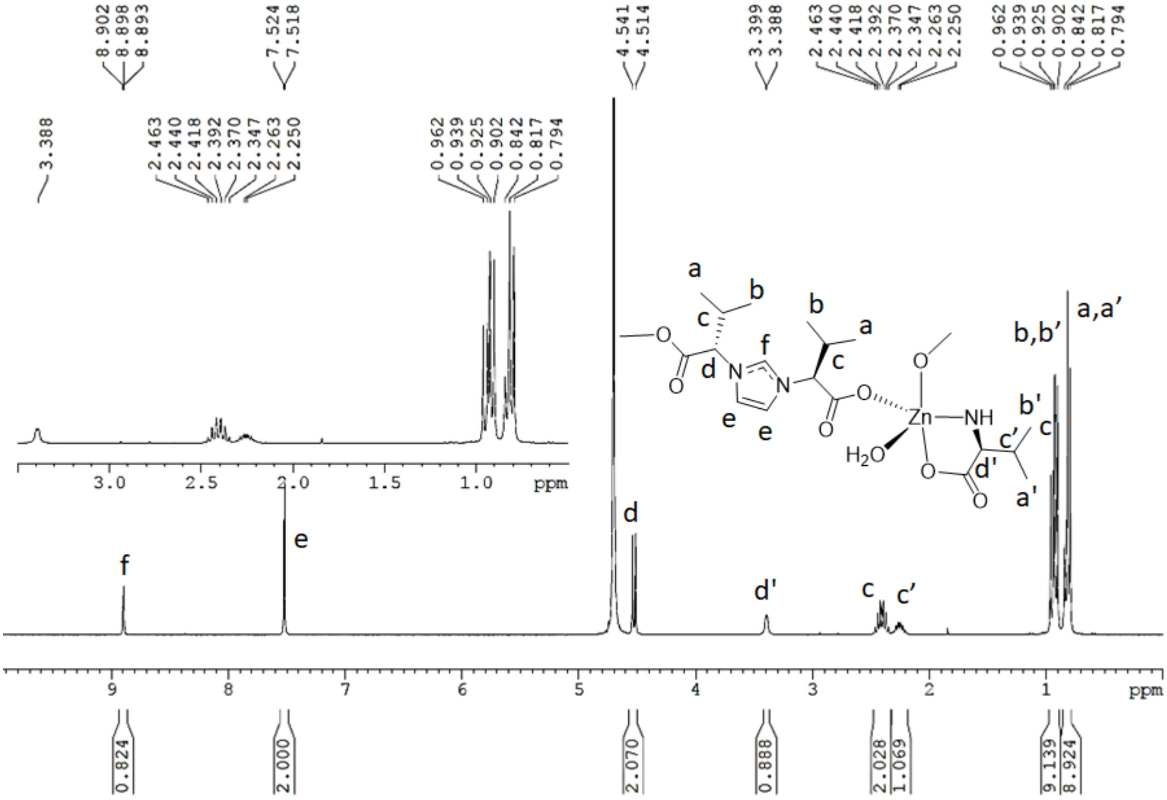

a,a'
b,b'
f
e
d
d'
c c'
ppm
0.824
2.000
2.070
0.888
2.028
1.069
9.139
8.924

2e

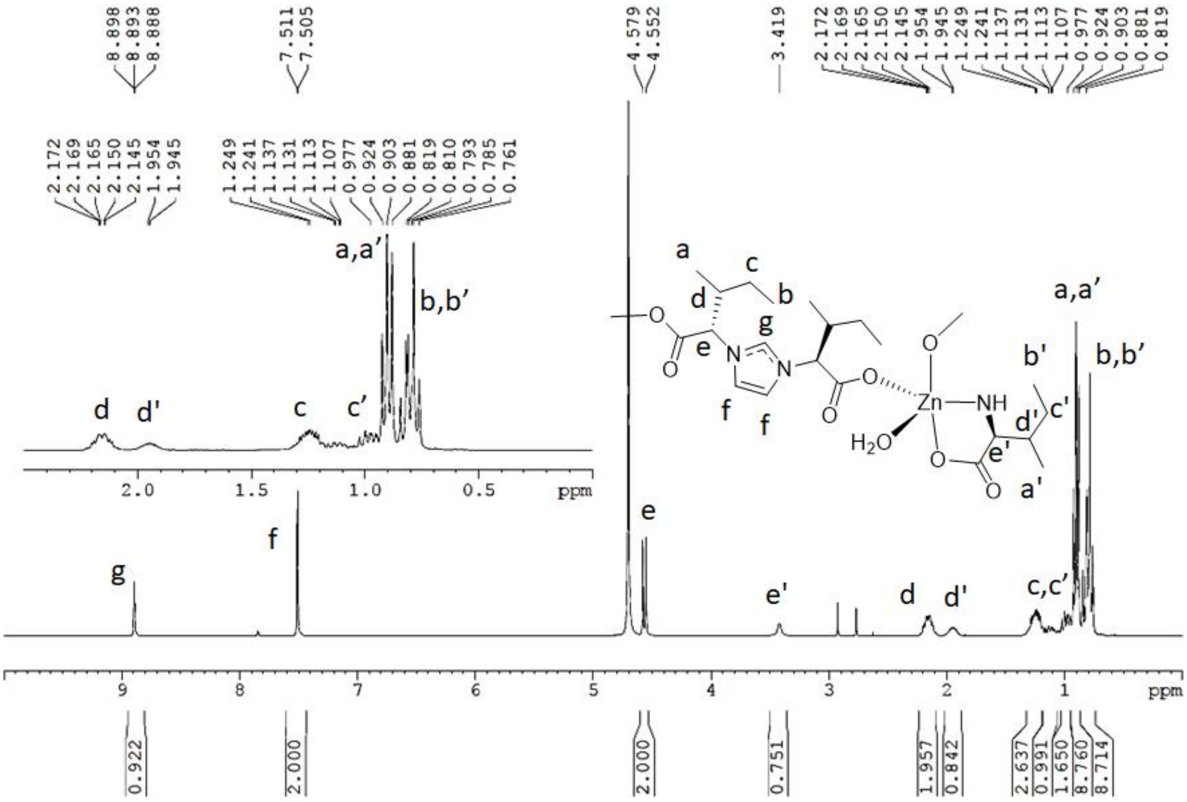

a,a'
b,b'
d d'
c c'
g
f
e
e'
c,c'
ppm
0.922
2.000
2.000
0.751
1.957
0.842
2.637
0.991
1.650
8.760
8.714

$^{13}C\{^{1}H\}$ *NMR spectra*

2a

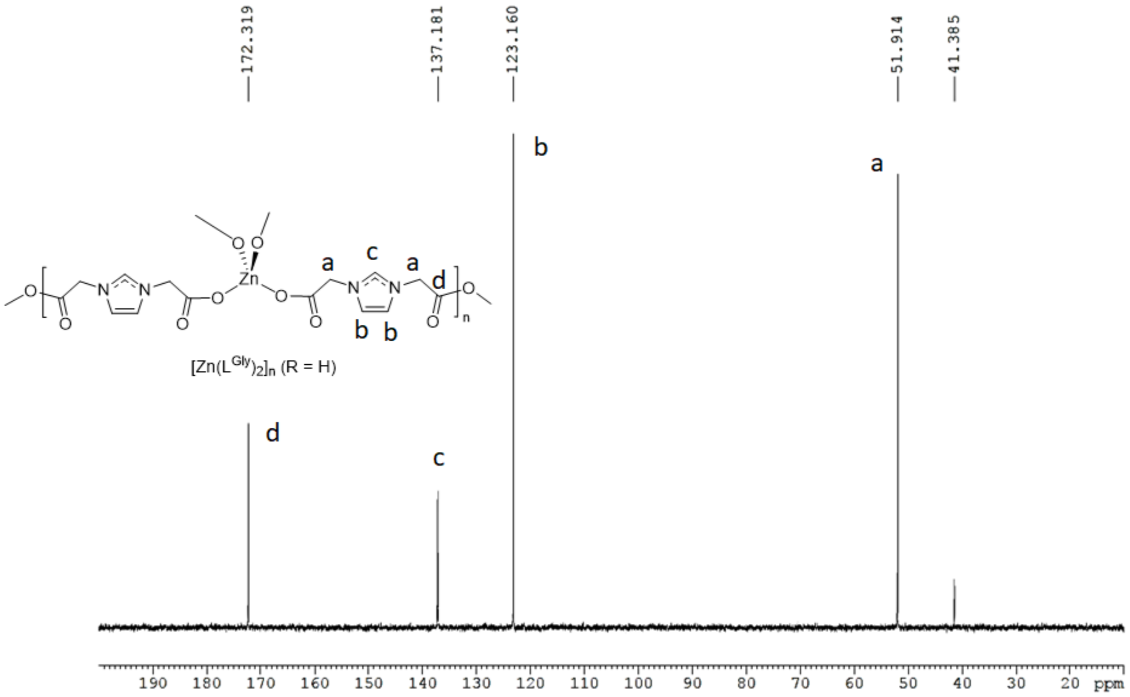

2b

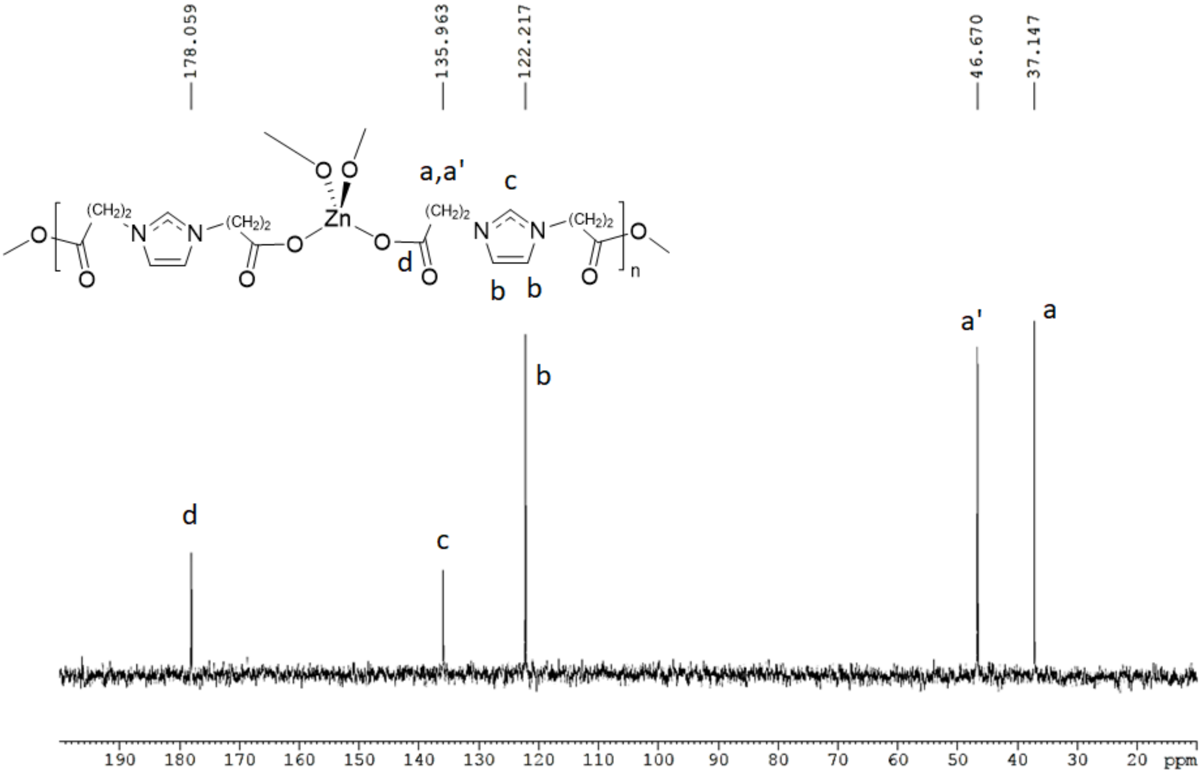

2c

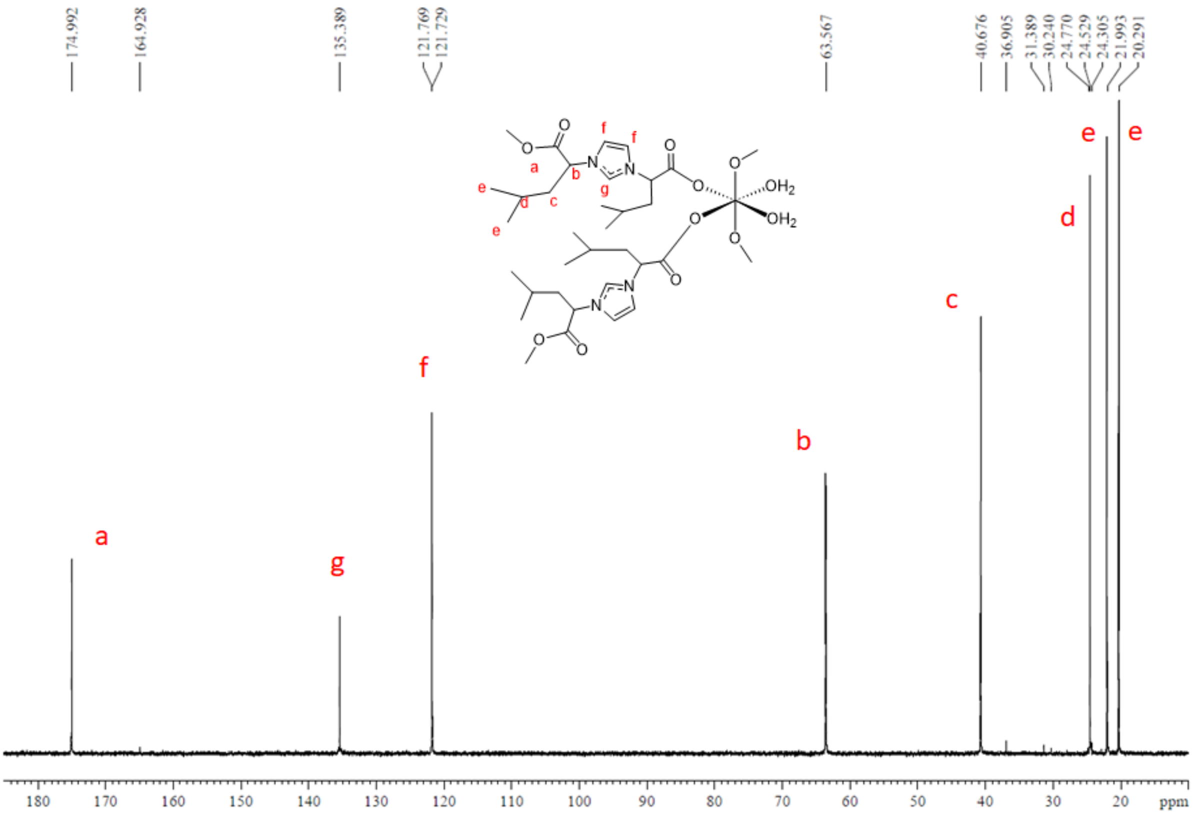
174.992
164.928
135.389
121.769
121.729
63.567
40.676
36.905
31.389
30.240
24.770
24.529
24.305
21.993
20.291
a
g
f
b
c
d
e
e
OH2
OH2
180
170
160
150
140
130
120
110
100
90
80
70
60
50
40
30
20
ppm

2d

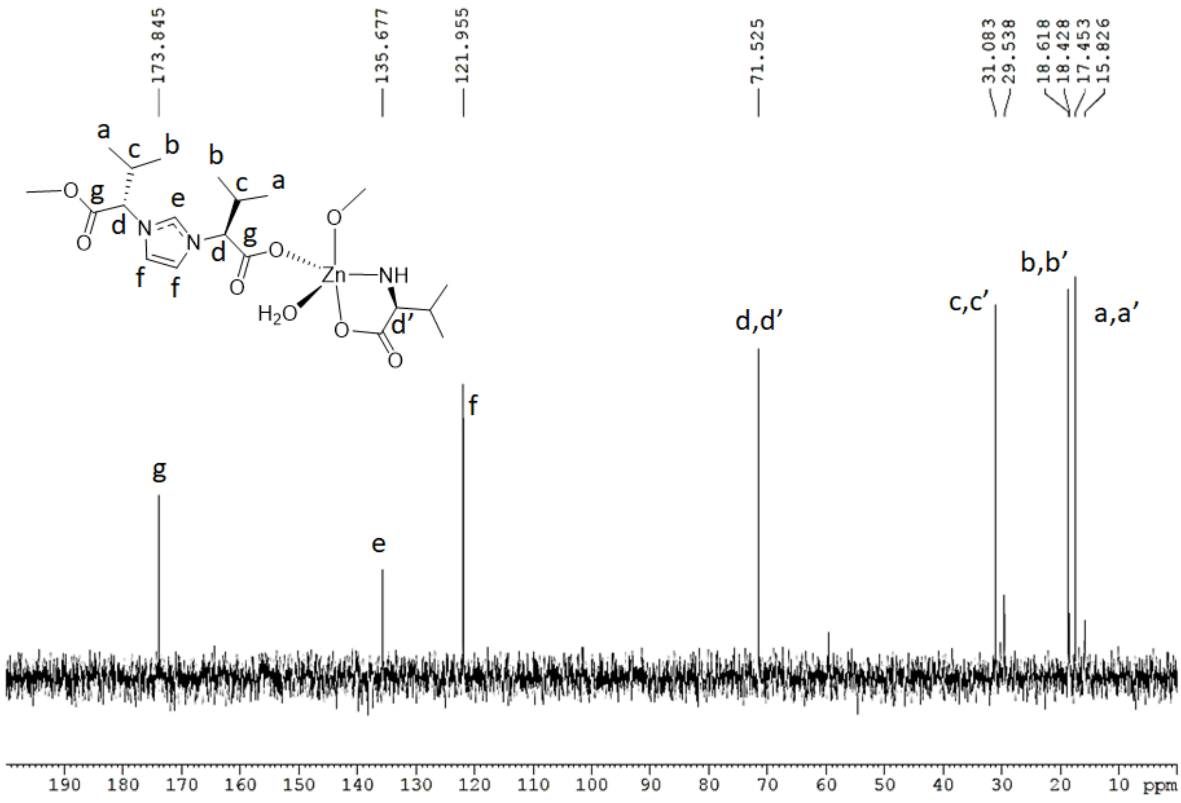
173.845
135.677
121.955
71.525
31.083
29.538
18.618
18.428
17.453
15.826
g
e
f
d,d'
c,c'
b,b'
a,a'
Zn
NH
H2O
190
180
170
160
150
140
130
120
110
100
90
80
70
60
50
40
30
20
10
ppm

2e

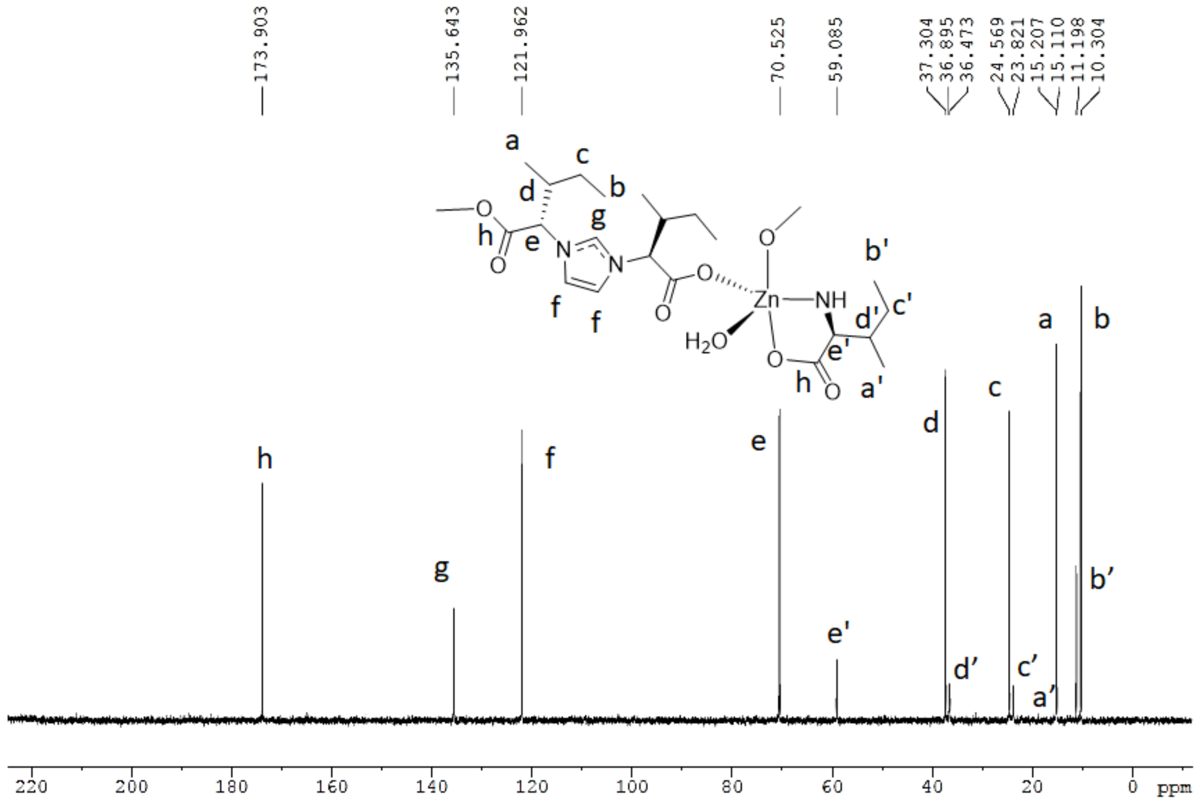
173.903
135.643
121.962
70.525
59.085
37.304
36.895
36.473
24.569
23.821
15.207
15.110
11.198
10.304
h
g
f
e
e'
d
d'
c
c'
a
a'
b
b'
Zn
NH
H_2O
220
200
180
160
140
120
100
80
60
40
20
0
ppm

Figure S2. Non-classical C=O···H-C hydrogen bonds observed between adjacent 1D chains in **2b**.

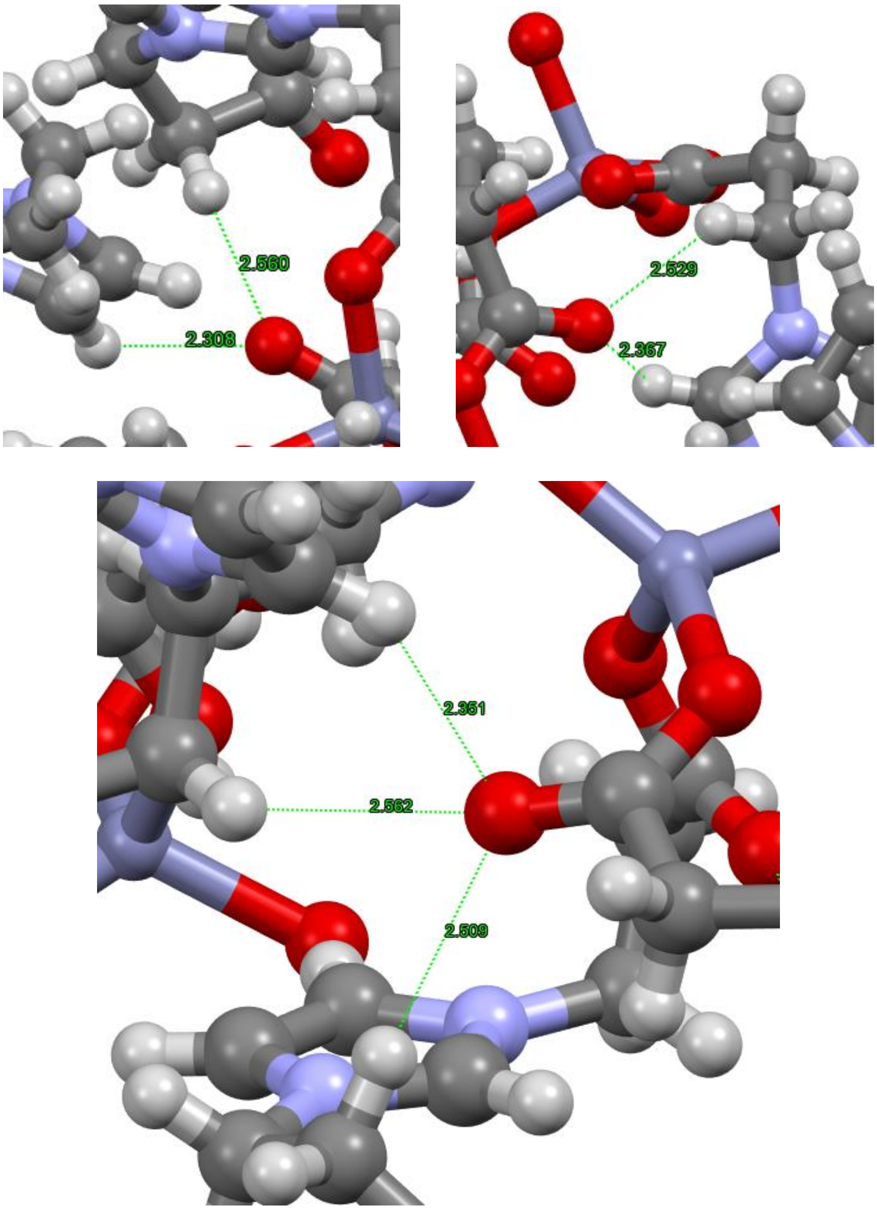

Figure S3. 3D crystal packing of complex **2b** viewed along *b* axis.

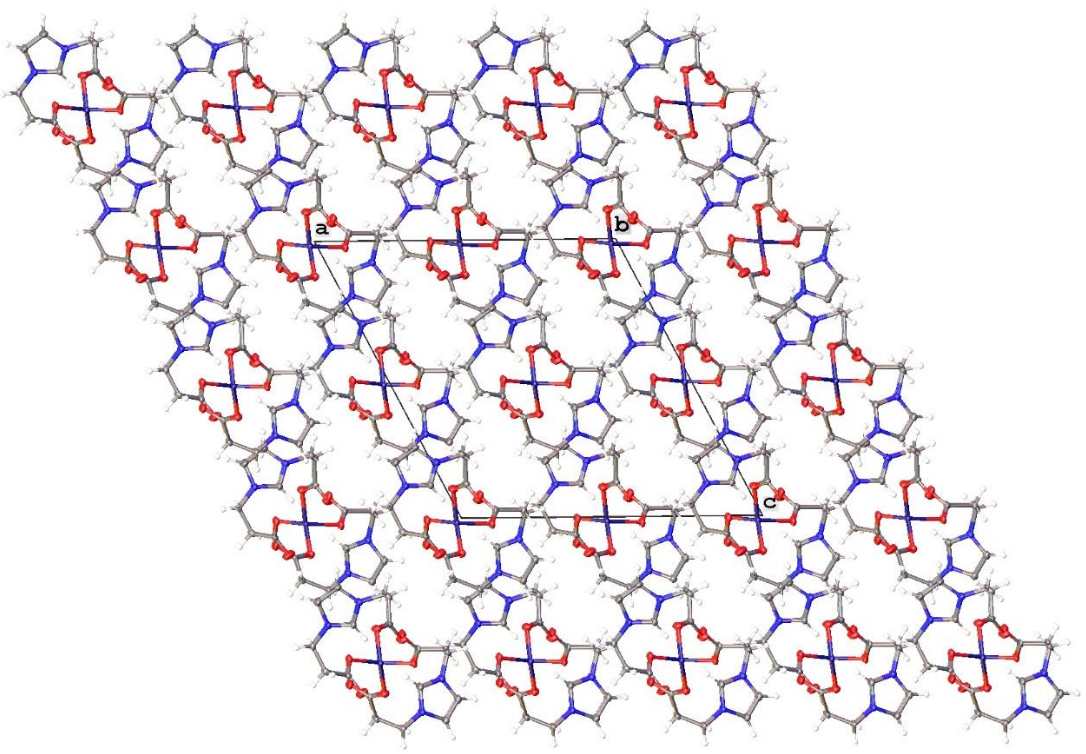

Figure S3. Square lattice topology of complex **2c**.

Figure S4. Complex **2c**: (a) 2D metal–organic framework; (b) 3D packing viewed along *a* axis; (c) square lattice topology.

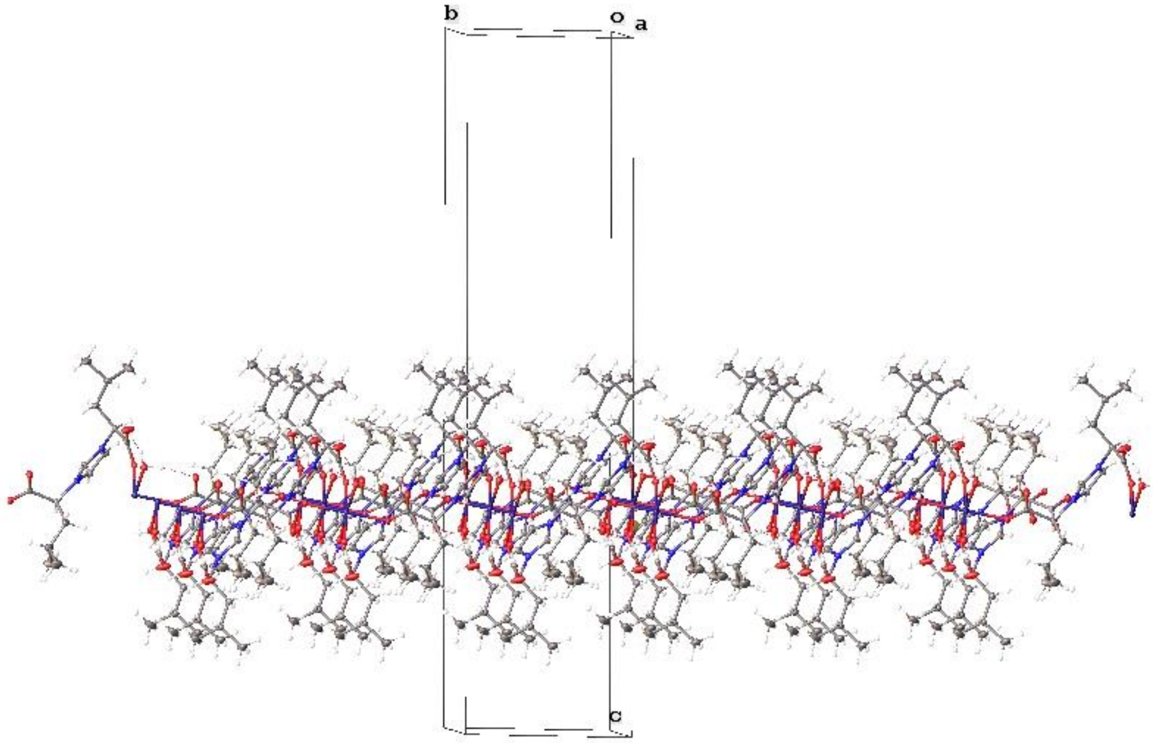

(a)

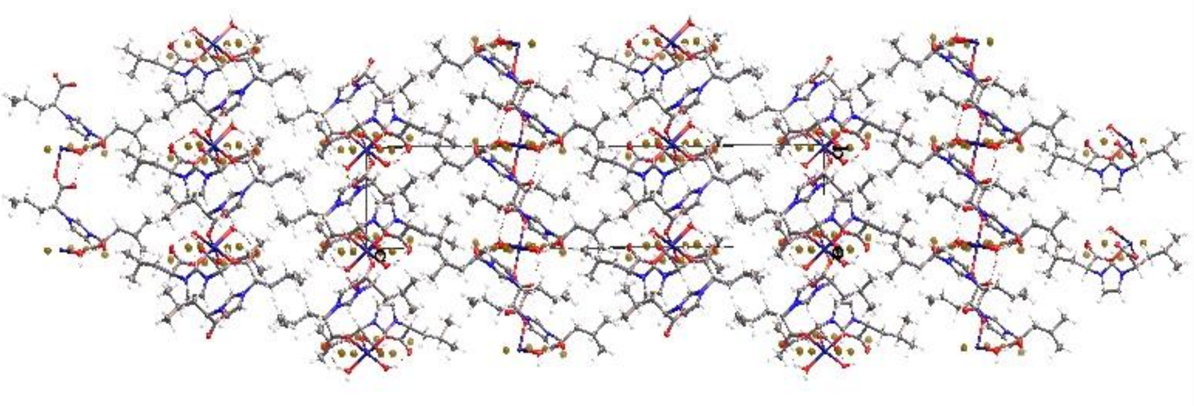

(b)

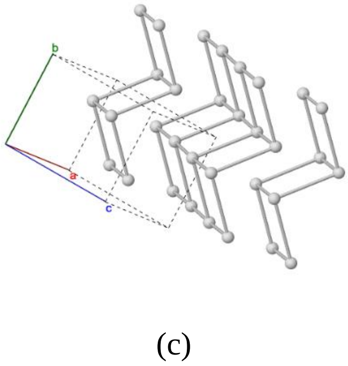

(c)

Figure S5. Complex **2d**: (a) 1D coordination polymer along *b* axis viewed along *a* axis. (b) 3D packing viewed along *b* axis.

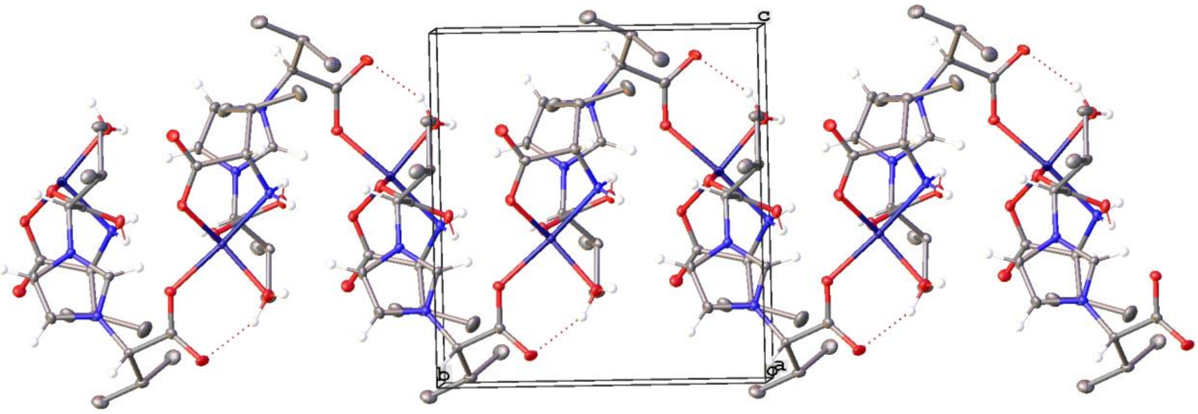

(a)

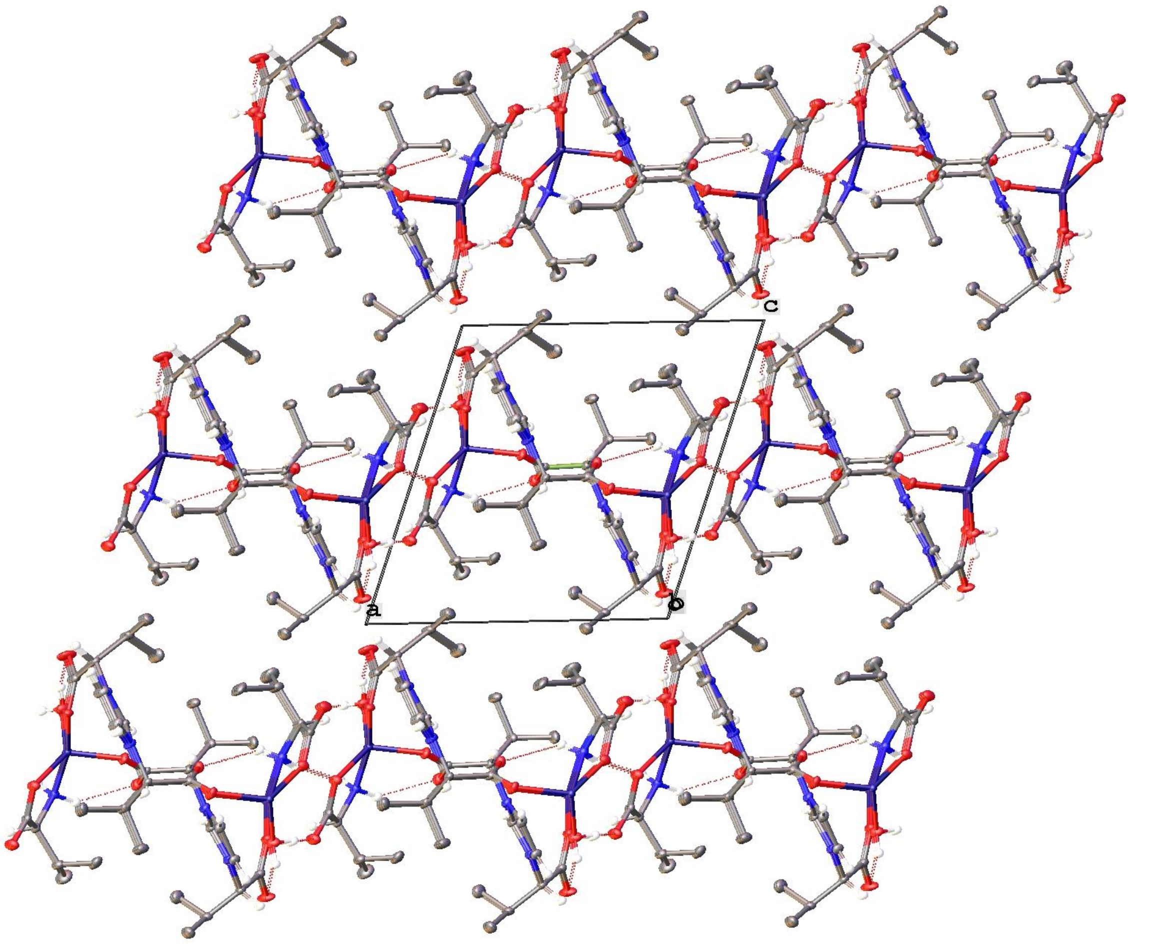

(b)

Table S1. Crystal data and structure refinement for complexes **2a-e**.

Complex	**2a**	
Empirical formula	$C_{14}H_{14}N_4O_8Zn$	
Formula weight	431.66	
Temperature	193(2) K	
Wavelength	0.71073 Å	
Crystal system	Monoclinic	
Space group	C2/c	
Unit cell dimensions	a = 17.1975(8) Å	α = 90°.
	b = 7.4004(3) Å	β = 106.583(2)°.
	c = 12.7633(6) Å	γ = 90°.
Volume	1556.80(12) $Å^3$	
Z	4	
Density (calculated)	1.842 g/cm^3	
Absorption coefficient	1.636 mm^{-1}	
F(000)	880	
Crystal size	0.300 x 0.100 x 0.050 mm^3	
Theta range for data collection	2.471 to 25.247°.	
Index ranges	$-20 \leq h \leq 20$, $-8 \leq k \leq 8$, $-15 \leq l \leq 15$	
Reflections collected	11319	
Independent reflections	1386 [R(int) = 0.0245, R_{sigma} = 0.0176]	
Completeness to theta = 25.242°	97.9 %	
Absorption correction	Semi-empirical from equivalents	
Max. and min. transmission	0.7461 and 0.6323	
Refinement method	Full-matrix least-squares on F^2	
Data / restraints / parameters	1386 / 0 / 123	
Goodness-of-fit on F^2	1.091	
Final R indices [I>2sigma(I)]	R1 = 0.0199, wR2 = 0.0498	
R indices (all data)	R1 = 0.0199, wR2 = 0.0499	
Extinction coefficient	n/a	
Largest diff. peak and hole	0.30 and -0.20 $e \cdot Å^{-3}$	

Complex	**2b**	
Empirical formula	$C_{18}H_{22}N_4O_8Zn$	
Formula weight	487.76	
Temperature	193(2) K	
Wavelength	0.71073 Å	
Crystal system	Monoclinic	
Space group	Cc	
Unit cell dimensions	a = 15.7484(11) Å	α= 90°.
	b = 8.6071(6) Å	β= 117.439(2)°.
	c = 16.3698(9) Å	γ = 90°.
Volume	1969.3(2) $Å^3$	
Z	4	
Density (calculated)	1.645 g/cm^3	
Absorption coefficient	1.304 mm^{-1}	
F(000)	1008	
Crystal size	0.500 x 0.300 x 0.100 mm^3	
Theta range for data collection	5.558 to 50.498°.	
Index ranges	$-18 \leq h \leq 18$, $-10 \leq k \leq 10$, $-17 \leq l \leq 19$	
Reflections collected	15938	
Independent reflections	3352 [R(int) = 0.0541, R_{sigma} = 0.0489]	
Completeness to theta = 25.242°	99.3 %	
Absorption correction	Semi-empirical from equivalents	
Max. and min. transmission	0.7461 and 0.2560	
Refinement method	Full-matrix least-squares on F^2	
Data / restraints / parameters	3352 / 2 / 281	
Goodness-of-fit on F^2	1.252	
Final R indices [I>2sigma(I)]	R1 = 0.0468, wR2 = 0.1013	
R indices (all data)	R1 = 0.0468, wR2 = 0.1014	
Absolute structure parameter	0.434(16)	
Extinction coefficient	n/a	
Largest diff. peak and hole	1.70 and -0.71 $e \cdot Å^{-3}$	

Complex	**2c**	
Empirical formula	$C_{30}H_{50}N_4O_{10}Zn$	
Formula weight	692.11	
Temperature	193(2) K	
Wavelength	0.71073 Å	
Crystal system	Trigonal	
Space group	$P3_221$	
Unit cell dimensions	a = 9.1488(2) Å	α= 90°.
	b = 9.1488(2) Å	β= 90°.
	c = 35.5558(15) Å	γ = 120°.
Volume	2577.33(16) $Å^3$	
Z	3	
Density (calculated)	1.338 g/cm^3	
Absorption coefficient	0.773 mm^{-1}	
F(000)	1104	
Crystal size	0.350 x 0.200 x 0.100 mm^3	
Theta range for data collection	5.142 to 50.47°.	
Index ranges	$-10 \leq h \leq 10$, $-10 \leq k \leq 10$, $-42 \leq l \leq 42$	
Reflections collected	31896	
Independent reflections	3071 [R(int) = 0.0367, R_{sigma} = 0.0233]	
Completeness to theta = 25.235°	99.9 %	
Absorption correction	Semi-empirical from equivalents	
Max. and min. transmission	0.7461 and 0.6462	
Refinement method	Full-matrix least-squares on F^2	
Data / restraints / parameters	3071 / 24 / 210	
Goodness-of-fit on F^2	1.009	
Final R indices [I>2sigma(I)]	R1 = 0.0264, wR2 = 0.0687	
R indices (all data)	R1 = 0.0267, wR2 = 0.0689	
Extinction coefficient	n/a	
Largest diff. peak and hole	0.64 and -0.49 $e \cdot Å^{-3}$	
Flack parameter	0.077(15)	

Complex	**2d**
Empirical formula	$C_{18}H_{31}N_3O_7Zn$
Formula weight	466.83
Temperature/K	193.00
Crystal system	monoclinic
Space group	$P2_1$
a/Å	10.6171(11)
b/Å	9.6392(10)
c/Å	11.0506(12)
α/°	90
β/°	108.895(4)
γ/°	90
Volume/Å^3	1070.0(2)
Z	2
ρ_{calc} /g·cm^{-3}	1.449
μ/mm^{-1}	1.191
F(000)	492.0
Crystal size/mm^3	0.5 × 0.12 × 0.1
Radiation	MoKα (λ = 0.71073)
2Θ range for data collection/°	5.858 to 50.49
Index ranges	$-12 \le h \le 12$, $-11 \le k \le 11$, $-13 \le l \le 13$
Reflections collected	12093
Independent reflections	3699 [R_{int} = 0.0471, R_{sigma} = 0.0504]
Data/restraints/parameters	3699/1/270
Goodness-of-fit on F^2	1.061
Final R indexes [I>=2σ (I)]	R_1 = 0.0286, wR_2 = 0.0732
Final R indexes [all data]	R_1 = 0.0314, wR_2 = 0.0748
Largest diff. peak/hole / e·Å^{-3}	0.45/-0.39
Flack parameter	0.109(14)

Complex	**2e**	
Empirical formula	$C_{21}H_{34}N_3O_7Zn$	
Formula weight	505.88	
Temperature	193(2) K	
Wavelength	0.71073 Å	
Crystal system	Monoclinic	
Space group	C_2	
Unit cell dimensions	a = 27.801(4) Å	α= 90°.
	b = 9.8432(10) Å	β= 108.040(6)°.
	c = 10.9492(14) Å	γ = 90°.
Volume	2848.9(6) Å^3	
Z	4	
Density (calculated)	1.179 Mg/m^3	
Absorption coefficient	0.900 mm^{-1}	
F(000)	1068	
Crystal size	0.500 x 0.300 x 0.100 mm^3	
Theta range for data collection	1.956 to 25.248°.	
Index ranges	$-33 \leq h \leq 33$, $-11 \leq k \leq 11$, $-13 \leq l \leq 13$	
Reflections collected	45207	
Independent reflections	5107 [R(int) = 0.0424]	
Completeness to theta = 25.242°	99.9 %	
Absorption correction	Semi-empirical from equivalents	
Max. and min. transmission	0.7461 and 0.6606	
Refinement method	Full-matrix least-squares on F	
Data / restraints / parameters	5107 / 221 / 341	
Goodness-of-fit on F^2	1.160	
Final R indices [I>2sigma(I)]	R1 = 0.0859, wR2 = 0.2531	
R indices (all data)	R1 = 0.0879, wR2 = 0.2560	
Absolute structure parameter	0.075(6)	
Extinction coefficient	n/a	
Largest diff. peak and hole	1.714 and -1.397 e·Å^{-3}	

Table S2. Selected structural parameters (bond lengths, Å; angles, °) for zinc complexes **2**.[a]

**Lengths**	**2a**	**2b**	**2c**	**2d**	**2e**
Zn-O	Zn(1)-O(1)#1 1.9539(11)<br>Zn(1)-O(1)#2 1.9539(11)<br>Zn(1)-O(4) 1.9556(11)<br>Zn(1)-O(4)#3 1.9556(11)	Zn(1)-O(2) 1.964(4)<br>Zn(1)-O(7) 1.968(4)<br>Zn(1)-O(6)#1 1.974(4)<br>Zn(1)-O(4)#1 1.977(4)	Zn(1)-O(1) 2.0506(18)<br>Zn(1)-O(3)#2 2.1790(18)<br>Zn(1)-O(5) 2.1179(18)	Zn(1)-O(2) 2.022(2)<br>Zn(1)-O(4)#1 2.068(3)<br>Zn(1)-O(6) 2.021(3)<br>Zn(1)-O(7) 2.032(3)	Zn(1)-O(4) 1.992(7)<br>Zn(1)-O(7) 2.039(9)<br>Zn(1)-O(6) 2.026(7)<br>Zn(1)-O(2)#1 2.064(8)
Zn-N	-	-	-	Zn(1)-N(3) 2.106(3)	Zn(1)-N(3) 2.110(8)
C-O	O(1)-C(5) 1.2838(19)<br>O(2)-C(5) 1.2234(19)<br>O(3)-C(7) 1.224(2)<br>O(4)-C(7) 1.2765(19)	O(1)-C(6) 1.219(7)<br>O(2)-C(6) 1.268(7)<br>O(3)-C(9) 1.234(8)<br>O(4)-C(9) 1.275(7)<br>O(5)-C(15) 1.213(8)<br>O(6)-C(15) 1.276(7)<br>O(7)-C(18) 1.260(7)<br>O(8)-C(18) 1.235(7)	O(1)-C(5) 1.254(3)<br>O(2)-C(5) 1.237(3)<br>O(3)-C(11) 1.252(3)<br>O(4)-C(11) 1.248(3)	O(1)-C(5) 1.234(4)<br>O(2)-C(5) 1.274(4)<br>O(3)-C(10) 1.233(5)<br>O(4)-C(10) 1.274(5)<br>O(5)-C(15) 1.238(5)<br>O(6)-C(15) 1.272(5)	O(1)-C(5) 1.249(14)<br>O(2)-C(5) 1.233(13)<br>O(3)-C(11) 1.260(14)<br>O(4)-C(11) 1.258(13)<br>O(5)-C(16) 1.239(13)<br>O(6)-C(16) 1.264(13)
**Angles**					
O-Zn-O	O(1)#1-Zn(1)-O(1)#2106.38(7)<br>O(1)#1-Zn(1)-O(4) 112.75(5)<br>O(1)#2-Zn(1)-O(4) 115.32(5)<br>O(1)#1-Zn(1)-O(4)#3115.32(5)<br>O(1)#2-Zn(1)-O(4)#3112.75(5)<br>O(4)-Zn(1)-O(4)#3 94.37(7)	O(2)-Zn(1)-O(7) 119.83(16)<br>O(2)-Zn(1)-O(6)#1 120.53(17)<br>O(7)-Zn(1)-O(6)#1 96.21(17)<br>O(2)-Zn(1)-O(4)#1 95.83(16)<br>O(7)-Zn(1)-O(4)#1 106.22(17)<br>O(6)#1-Zn(1)-O(4)#1 118.93(17)	O(1)#1-Zn(1)-O(1) 90.57(10)<br>O(1)-Zn(1)-O(5)#1 176.28(9)<br>O(1)-Zn(1)-O(5) 89.68(7)<br>O(5)#1-Zn(1)-O(5) 90.31(11)<br>O(1)-Zn(1)-O(3)#2 85.38(7)<br>O(5)-Zn(1)-O(3)#2 90.70(7)<br>O(1)-Zn(1)-O(3)#3 93.02(7)<br>O(5)-Zn(1)-O(3)#3 90.90(7)<br>O(3)#2-Zn(1)-O(3)#3 177.73(10)	O(2)-Zn(1)-O(7) 103.31(12)<br>O(2)-Zn(1)-O(4)#1 88.66(11)<br>O(2)-Zn(1)-O(6) 116.06(11)<br>O(6)-Zn(1)-O(4)#1 88.18(10)<br>O(6)-Zn(1)-O(7) 140.43(13)<br>O(7)-Zn(1)-O(4)#1 89.00(11)	O(4)-Zn(1)-O(7) 102.3(4)<br>O(4)-Zn(1)-O(6) 117.1(3)<br>O(7)-Zn(1)-O(6) 140.5(4)<br>O(4)-Zn(1)-O(2)#1 88.7(3)<br>O(7)-Zn(1)-O(2)#1 89.4(3)<br>O(6)-Zn(1)-O(2)#1 89.0(3)
O-Zn-N	-	-	-	O(2)-Zn(1)-N(3) 103.08(11)<br>O(4)#1-Zn(1)-N(3)166.58(11)<br>O(6)-Zn(1)-N(3) 80.88(11)<br>O(7)-Zn(1)-N(3) 94.48(13)	O(4)-Zn(1)-N(3) 103.0(3)<br>O(7)-Zn(1)-N(3) 93.1(4)<br>O(6)-Zn(1)-N(3) 81.1(3)<br>O(2)#1-Zn(1)-N(3) 167.2(3)
O-C-O	O(2)-C(5)-O(1) 127.35(15)<br>O(3)-C(7)-O(4) 126.70(15)<br>O(3)-C(7)-C(6) 120.10(14)<br>O(4)-C(7)-C(6) 113.12(14)	O(1)-C(6)-O(2) 126.1(5)<br>O(3)-C(9)-O(4) 125.6(5)<br>O(5)-C(15)-O(6) 124.4(6)<br>O(8)-C(18)-O(7) 126.1(5)	O(2)-C(5)-O(1) 127.6(3)<br>O(4)-C(11)-O(3) 127.3(3)	O(1)-C(5)-O(2) 126.7(3)<br>O(3)-C(10)-O(4) 126.8(4)<br>O(5)-C(15)-O(6) 124.6(4)	O(2)-C(5)-O(1) 126.7(10)<br>O(3)-C(11)-O(4) 125.6(8)<br>O(5)-C(16)-O(6) 126.1(9)

a Symmetry transformations used to generate equivalent atoms: complex **2a**: #1 x+1/2, y+1/2, z. #2 -x+3/2, y+1/2, -z+1/2. #3 -x+2,y,-z+1/2, #4 x-1/2, y-1/2, z. complex **2b**: #1 x, y-1, z. #2 x, y+1, z. Complex **2c**: #1 x-y, -y, -z+4/3. #2 x, y-1, z. #3 x-y+1, -y+1, -z+4/3. #4 x, y+1, z. Complex **2d**: #1 1-x, 1/2+y, 1-z. Complex **2e**: #1 -x+3/2, y-1/2, -z+1. #2 -x+3/2, y+1/2, -z+1.

Figure S6. Complex **2a**: (a) asymmetric unit of complex, (b) polymeric growing, (c) 3D packing viewed along *b* axis, (d) 3D packing viewed along *a* axis.

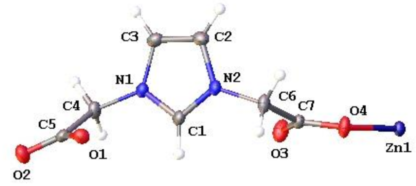

(a)

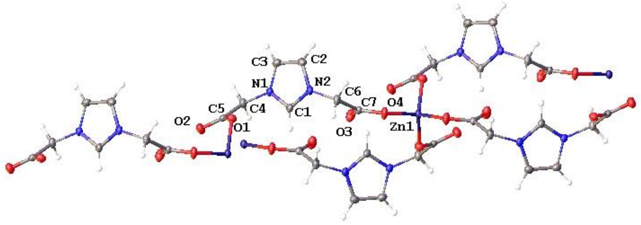

(b)

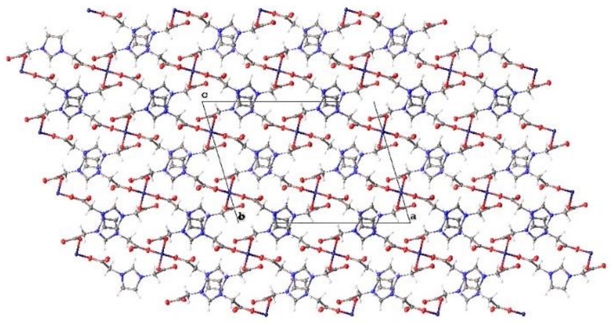

(c)

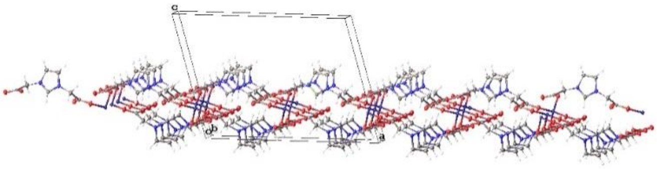

(d)

Table S3. IC_{50} values (μM) and selectivity indexes (SI) of common anticancer drugs tested against human cell lines.[a]

Compound	IC50 (Mean ± SEM) (Selectivity Index, Mean ± SEM)			
	**HaCaT** (Non-malignant keratinocyte)	**A549** (Lung adenocarcinoma)	**MeWo** (Melanoma)	**T24** (Bladder cancer)
Carboplatin (μM)	11.4 ± 1.0	21.1 ± 4.0 (0.6 ± 0.2)	23.0 ± 5.5 (0.6 ± 0.2)	7.3 ± 1.1 (1.7 ± 0.3)
Cisplatin (μM)	2.2 ± 0.5	3.8 ± 0.1 (0.6 ± 0.1)	2.2 ± 0.6 (1.0 ± 0.0)	1.6 ± 0.4 (1.3 ± 0.0)
Sunitinib (μM)	1.0 ± 0.1	1.5 ± 0.7 (1.2 ± 0.6)	0.9 ± 0.4 (1.5 ± 0.5)	1.9 ± 1.1 (1.5 ± 0.8)
5-Fluorouracil (μM)	0.4 ± 0.0	1.9 ± 0.7 (0.3 ± 0.1)	1.3 ± 0.0 (0.3 ± 0.0)	6.3 ± 0.6 (0.1 ± 0.0)
Gemcitabine (nM)	10.6 ± 4.3	1.9 ± 1.5 (5.1 ± 0.3)	1.9 ± 1.5 (5.1 ± 0.2)	1.7 ± 1.5 (6.5 ± 1.4)
Doxorubicin (nM)	8.1 ± 0.0	38.4 ± 6.6 (0.2 ± 0.0)	15.3 ± 3.6 (0.6 ± 0.1)	29.8 ± 2.0 (0.3 ± 0.0)
Paclitaxel (nM)	0.3 ± 0.0	3.5 ± 0.9 (0.1 ± 0.0)	0.7 ± 0.0 (0.5 ± 0.0)	1.4 ± 0.3 (0.3 ± 0.1)

[a]After 96 h of treatment, cell viability was measured using the resazurin assay. IC_{50} values are given as the mean value obtained from at least three independent experiments ± standard error of the mean (SEM) (see Materials and Methods for more details). The selectivity index (SI) values were calculated as the average of the IC_{50} values in the HaCaT non-cancerous cell line divided by the IC_{50} value in the cancer cell line obtained in each independent experiment.

Figure S7. Effect of HL^R compounds **1** (R = Gly, βAla, Val, Leu, and Ile) on the viability of human non-malignant cells (HaCaT) and human cancer cells (A549, MeWo and T24).[¥]

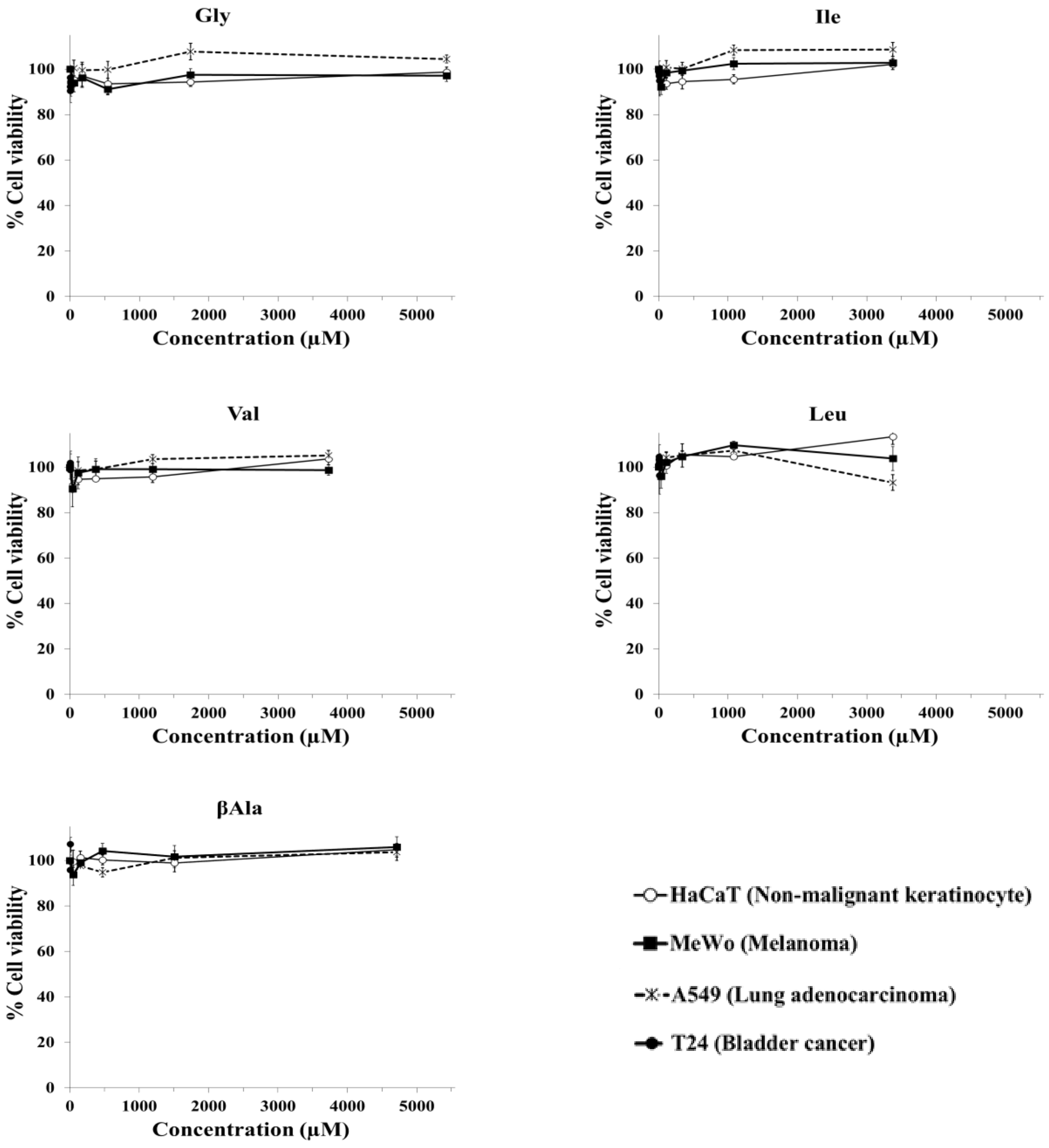

[¥] Cells were exposed to reagents for 72 hours and cell viability was measured using the resazurin assay. Data represent mean ± SEM from at least three independent experiments.

Figure S8. Representative photographs of untreated cells (control), cells treated with positive control carboplatin and gemcitabine, and cells exposed to complexes **2a** and **2c**. Cells were exposed to the drugs for 72 hours and cell viability was measured using the resazurin assay. Images were taken using a Huawei P9 lite Leica camera adapted to an inverted microscope (magnification 10x).

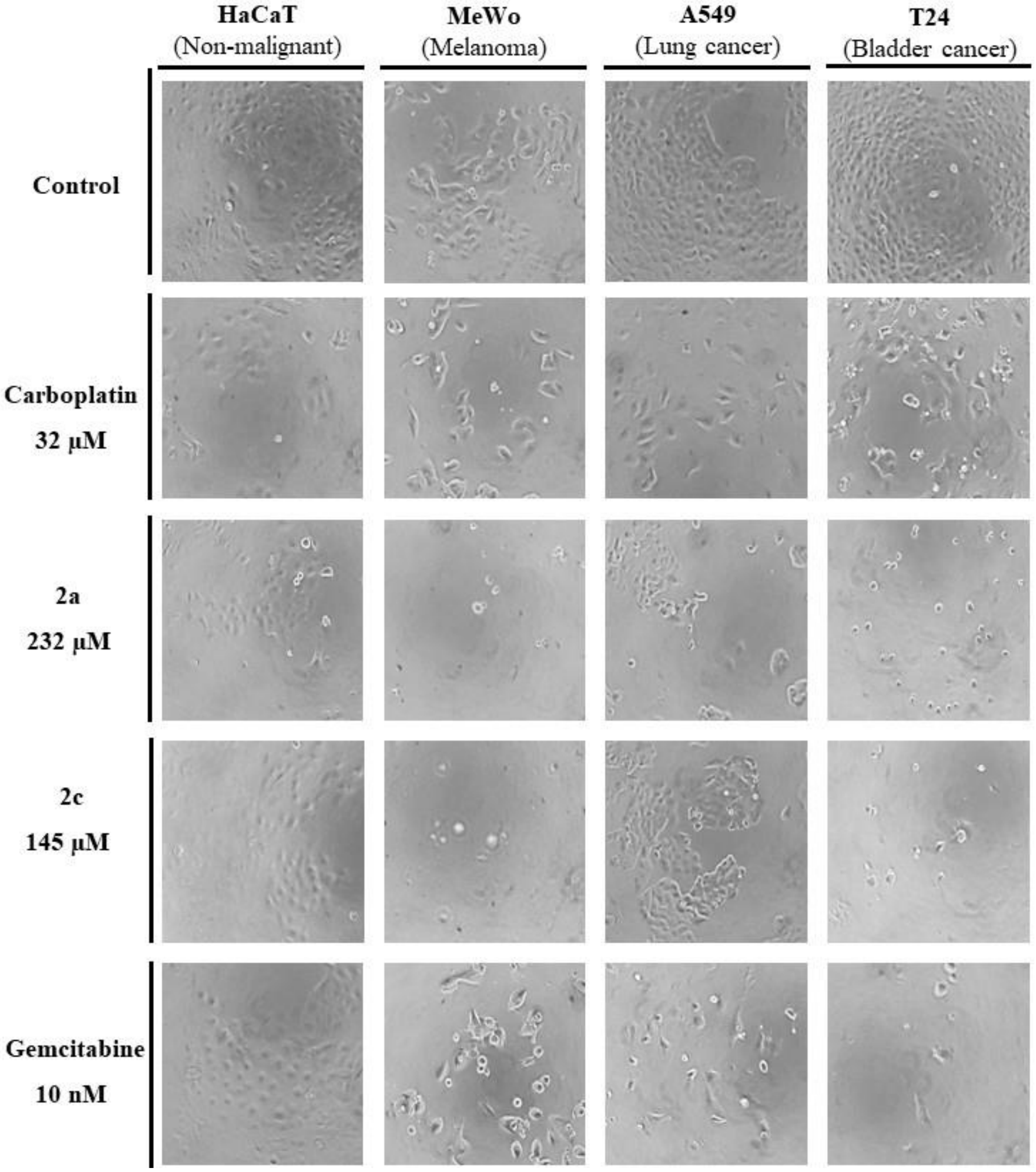

Supplement: Supplementary file 1 [file ijms-26-03202-s001.zip › SuppMat_Zn_IJMS_AAs_vf.pdf]
